# Associations Between Openness Facets, Prejudice, and Tolerance: A Scoping Review With Meta-Analysis

**DOI:** 10.3389/fpsyg.2021.707652

**Published:** 2021-09-28

**Authors:** D. X. Ng, Patrick K. F. Lin, Nigel V. Marsh, K. Q. Chan, Jonathan E. Ramsay

**Affiliations:** School of Social and Health Sciences, James Cook University, Singapore, Singapore

**Keywords:** openness to experience, prejudice, tolerance, attitudes, personality, trait, facet

## Abstract

The personality factor of openness to experience, which encompasses curiosity, imagination, and a desire for new experiences, has been associated negatively with prejudice and positively with the closely related value of tolerance. While these relationships have been reviewed at the factor level, there has been no review of research at the lower facet level. This review aims to uncover the relationships between the facets of openness and the constructs of prejudice and tolerance. We conducted a preregistered scoping review with meta-analysis following the recommended guidelines from Joanna Briggs Institute. A total of 2,349 articles were reviewed, with 16 primary research articles (or 17 studies) meeting the criteria for inclusion. Aggregated effect sizes via random-effect meta-analysis revealed that all revised neuroticism-extraversion-openness personality inventory (NEO-PI-R) and international personality item pool (IPIP)-based facets of openness significantly predicted prejudice and tolerance. Out of the three measures [i.e., NEO-PI-R, IPIP-NEO, and honesty-humility, emotionality, extraversion, agreeableness, conscientiousness, and openness to experience personality inventory (HEXACO-PI), and the facets of openness examined], the NEO-PI-R facet of value was most strongly associated with prejudice. In contrast, the NEO-PI-R facet of aesthetics was the facet most strongly associated with tolerance. However, these results should be treated as preliminary in light of the small number of meta-analyzed studies and more primary research studies are needed to confirm the trends found in this review. This review represents the first step in the systematic investigation of the link between the facets of openness and components of prejudice and tolerance and contributes toward explaining prejudice and tolerance.

## Introduction

Recent high-profile examples of prejudice, discrimination, and violence against ethnic minorities in the United States and elsewhere have reignited a global discourse on the causes of prejudice and possible solutions (Subbaraman, 2020). Prejudice, defined as a negative attitude toward others based on their social group membership (Allport, 1954), is prevalent worldwide (Duckitt, 2019). Prejudice manifests itself in various forms (Abrams, 2010; Liao et al., 2016). For instance, prejudice can be expressed explicitly (e.g., “I dislike immigrants”; Legault et al., 2007), implicitly (e.g., a strong latent reaction toward pairing negative words with immigrants; Legault et al., 2007), blatantly (e.g., “immigrants are generally not very intelligent”; Akrami et al., 2000), or subtly (e.g., “It is a matter of not trying hard enough, if immigrants would only try harder, they could be as well off as the locals”; Pettigrew and Meertens, 1995). Prejudice carries severe negative social implications. For instance, prejudice causes harm to the social fabric of society (like inciting intergroup hostility, reducing willingness to cooperate; Tropp, 2003; Noh et al., 2007; Williams, 2018) and causes devastating physical and mental health outcomes to the individuals who experience prejudice (like increased rates of cardiovascular disease, mortality, post-traumatic stress, substance abuse, depression, and suicidal attempts; Ashburn-Nardo et al., 2007; Harris et al., 2012; Paradies et al., 2015; Dover et al., 2020). This review offers an insight into the possible causes of prejudice by examining how prejudicial attitudes and values associated with tolerance associate with the personality factor of openness and its underlying facets.

### Openness to Experience and the Big Five Personality Model

**Figure 1:**
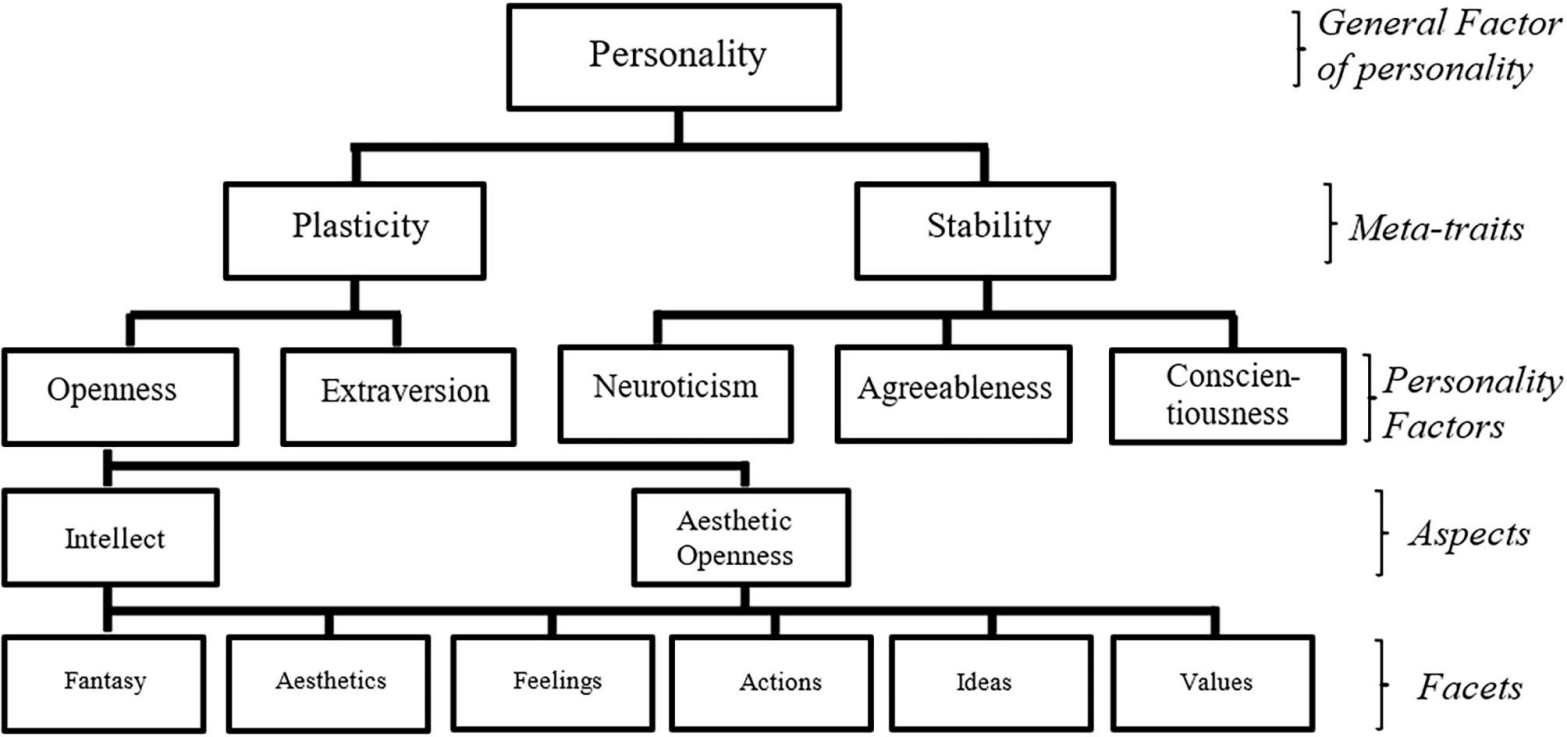
Hierarchical model of personality (Costa and McCrae, 1992; Digman, 1997; McCrae et al., 2005; DeYoung et al., 2007; Rushton and Irwing, 2008). The descriptors referring to each of the hierarchical levels are provided on the right of this figure.

Openness to experience, also known as the openness factor, is a major personality dimension of the Big Five personality model (Saucier and Ostendorf, 1999). Traditionally, the Big Five factors are numbered and termed as follows: (1) surgency (or extraversion), (2) agreeableness, (3) conscientiousness (or dependability), (4) emotional stability (as opposed to neuroticism), and (5) culture (Figure 1; Norman, 1963; Digman, 1990). However, different lexical studies have uncovered slightly different themes in their fifth factor, causing the fifth factor to be renamed as intellect (Goldberg, 1990) or openness (McCrae and Costa, 1987). Generally, the fifth factor has been associated with characteristics, such as being polished, refined, imaginative, reflective, and artistically sensitive (i.e., culture; Norman, 1963), possessing wisdom, originality, objectivity, and knowledge (i.e., intellect; Goldberg, 1990), or being imaginative, aesthetically inclined, attracted to variety, and liberal in values (i.e., openness; Boies et al., 2001; Costa and McCrae, 2008).

Personality, however, is not thought to be entirely unidimensional. Instead, personality psychologists have a consensus that personality exhibits a hierarchical structure, where higher-level personality factors subsume lower-level personality facets (Figure 1; Digman, 1990; Goldberg, 1993; Judge et al., 2013). For example, the openness factor is placed on a higher hierarchy level while openness facets, such as fantasy, aesthetics, feelings, actions, ideas, and values, are placed on a lower level (Figure 1; McCrae et al., 2005). The hierarchical structure of personality is well-validated (Digman, 1997; Mount et al., 2005; DeYoung, 2006; Rushton and Irwing, 2008; Woo et al., 2014a), with a general agreement that that there is one general factor of personality at the broadest and the highest level (Rushton and Irwing, 2008), two meta-traits (where plasticity refers to the basic tendencies toward personal growth, while stability refers to the basic tendencies toward socialization; Digman, 1997), followed by the Big Five factors (i.e., openness, conscientiousness, extraversion, agreeableness, and neuroticism), then aspects (DeYoung et al., 2007; Mussel et al., 2011), and lastly the facets at the lowest level of the hierarchy (Figure 1; McCrae et al., 2005).

Proponents of hierarchically structured personality propose that the openness factor accounts for broad behavioral tendencies while lower-level openness facets account for more specific behavioral inclinations (Judge et al., 2013). Several researchers have argued that personality facets may afford higher explanatory potential than personality factors as they contain specific variance that accounts for individual differences beyond those of the common factor (Ashton, 1998; Paunonen et al., 1999; Elleman et al., 2020). A recent meta-analysis supported this claim where they found regression models with all narrow facets combined outperformed models with all broad factors combined in the prediction of workplace behaviors (Pletzer et al., 2020). Similarly, several studies found openness facets held stronger associations with specific behavioral outcomes than the broad openness factor (Griffin and Hesketh, 2004; Hastings and O'Neill, 2009; Woo et al., 2014b). For instance, task performance was found significantly correlated with the openness facets of values (*r* = 0.49, *p* < 0.01; Griffin and Hesketh, 2004), intellect (*r* = 0.17, *p* < 0.05; Griffin and Hesketh, 2004), and ingenuity (*r* = 0.15, *p* < 0.05; Woo et al., 2014b) but not with the broad openness factor (*r* = 0.07, n.s.; Griffin and Hesketh, 2004; *r* = 0.05, n.s.; Woo et al., 2014b). All these findings supported the claim that openness facets improve the predictive validities of openness on behavioral outcomes.

Although there is little dispute on the hierarchical representation of personality (Judge et al., 2013), personality psychologists have disagreed on the number of facets underlying each personality factor (Hogan et al., 1996; Cattell and Mead, 2008). For instance, there are currently more than 10 different personality measures of openness facets, which exhibit significant variability in the number of facets underlying the openness factor (Schwaba et al., 2020). Openness factor has been variously proposed to comprise three (intellect, imaginative-creative, and perceptive; Saucier and Ostendorf, 1999), four (creative, unconventional, inquisitive, and aesthetic appreciation; Lee and Ashton, 2004), five (creative-uncreative, inquisitive-uninquisitive, deep-shallow, individualistic-dependent, and perceptive-unobservant; Hofstee et al., 1992), and six facets (fantasy, aesthetics, feelings, actions, ideas, and values; McCrae et al., 2005). The key reason for the lack of consensus is because personality theorists had adopted different approaches in their conceptualization of the underlying facet structure of the openness factor (Glick and Fiske, 1996; Hough and Ones, 2001; Woo et al., 2014a). The approaches adopted by personality theorists can be categorized as either the questionnaire approach (where the factor analyses of similar measures derive the facet structure, e.g., NEO-PI-R; Costa and McCrae, 1992) or the lexical approach [where the empirical reduction of personality-describing adjectives derives the facet structure; e.g., humility, emotionality, extraversion, agreeableness, and conscientiousness and openness to experience personality inventory (HEXACO-PI) by Lee and Ashton (2004), and IPIP-based measures by Goldberg (1999); see Table 1 for the definition of the facets of openness within revised neuroticism-extraversion-openness personality inventory (NEO-PI-R), HEXACO-PI, and international personality item pool (IPIP)-based measures]. These different approaches have resulted in many different conceptualizations of the openness factor and its constituent facets, further contributing to the status of the openness factor as one of the least understood personality constructs.

**Table 1:** Definition of openness facets in NEO-PI-R, HEXACO-PI, and IPIP-based measures.

**Openness facet**	**Description**	**Example item**
**NEO-PI-R/IPIP-based measures**		
Values (NEO-PI-R)/Liberalism (IPIP)	Readiness to challenge authority and reexamine values	“I believe that there is no absolute right or wrong”
Aesthetics (NEO-PI-R)/Artistic Interests (IPIP)	Appreciation of natural and artificial beauty	“I see beauty in things that others might not notice”
Feelings (NEO-PI-R)/Emotionality (IPIP)	Awareness of own's own inner feelings	“I feel others' emotions”
Fantasy (NEO-PI-R)/Imagination (IPIP)	Uses imagination to create an interesting inner world	“I have a vivid imagination”
Ideas (NEO-PI-R)/Intellect (IPIP)	Willingness to consider new and unusual ideas	“I am interested in abstract ideas”
Actions (NEO-PI-R)/Adventurousness (IPIP)	Eager to try new activities and experience new things	“I prefer variety to routine”
**HEXACO-PI**		
Aesthetic appreciation	Appreciation of beauty in arts and in nature	“I can spend a long time studying a painting that I like”
Inquisitiveness	Eager to experience all aspects of nature and human world	“I enjoy looking at maps of different places”
Creativity	Preference for originality and innovative	“I would enjoy creating a work of art”
Unconventionality	Willingness to accept the unusual	“I like hearing about opinions that are very different from those of most people”

*Adapted from Costa and McCrae (1992), Lee and Ashton (2004), and Maples et al. (2014)*.

To better understand the openness and its constituent facets, several researchers have argued for more research studies into the criterion-related validity of the openness facets (e.g., Hastings and O'Neill, 2009; Judge et al., 2013; Woo et al., 2014b; Christensen et al., 2019; Schwaba et al., 2020). Accumulating evidence on the predictor-criterion validity of narrow facets facilitates the understanding of the facet-specific variance that is often masked by aggregating facet scores into factor scores (Pletzer et al., 2020). In addition, identifying the differential criterion-relations of openness facets unveiled the facet-level relationship between openness and the outcome variable. For instance, job stress has been found to be uncorrelated with openness factor but was negatively correlated with the openness facets of liberalism (*r* = −0.15, *p* < 0.05) and adventurousness (*r* = −0.21, *p* < 0.01), and positively correlated with the openness facets of imagination (*r* = 0.25, *p* < 0.01) and emotionality (*r* = 0.29, *p* < 0.01; Griffin and Hesketh, 2004). In this review, we attempt to review the current evidence of the predictive utility of the facets of openness in a very different domain: that of prejudice and tolerance.

### Facets of Openness and Prejudice

Low openness individuals, characterized by black-or-white thinking style, intolerance of ambiguity, authoritarianism, dislike of change, and rejection of deviance from the social norms, have consistently been found to be more prejudiced than their more open-minded counterparts (Hodson and Dhont, 2015). Conversely, individuals high in openness have been consistently linked with lower prejudice (Flynn, 2005; Duriez and Soenens, 2006; Ekehammar and Akrami, 2007; Sibley and Duckitt, 2008; Sturmer et al., 2013). Sibley and Duckitt (2008) conducted a meta-analysis of 71 studies. They reported that the openness factor had the strongest association with prejudice toward low status and disadvantaged groups like illegal immigrants, African Americans, female, and Asian immigrants out of the Big Five factors. Specifically, high openness individuals were found to be less prejudiced toward low-status outgroups. These findings were corroborated by Crawford and Brandt (2019), where their meta-analysis found high openness individuals were less prejudiced toward mixed-status outgroups (e.g., Mormons, rich people, atheists, antigay activists, bankers, Evangelical Christians, and conservatives).

Although the openness factor has a consistent negative relationship with prejudice, the strength of this association does not appear to be consistent across measures. For instance, in their meta-analytic study, Sibley and Duckitt (2008) found a significant difference in the correlation between the openness factor and prejudice across different personality measures. In their study, the openness factor was strongly correlated with prejudice when measured with the NEO-PI-R (Costa and McCrae, 2008) but weakly associated with prejudice when measured with the Big Five Inventory (BFI; John and Srivastava, 1999). One possible explanation is that NEO-PI-R captured both the factor-level and facet-level variance of openness associated with prejudice, while the BFI only captured the factor-level variance of openness (Sibley and Duckitt, 2008). Another explanation is that each personality measure captures a different subset of openness facets that may or may not relate to prejudice. To understand the role of openness in prejudice and tolerance, we argue that examining the specific contributions of a wide range of openness facets, as operationalized by various leading personality measures, might provide a more nuanced understanding.

### Facets of Openness and Tolerance

Tolerance, defined as a value orientation toward difference (Hjerm et al., 2019), offers social psychologists an avenue to examine positive intergroup relations and the positive aspects of intergroup perceptions (Butrus and Witenberg, 2013). However, tolerance research has been made complicated by the fact that many researchers have treated prejudice and tolerance as if they were opposite ends of a spectrum (Verkuyten and Slooter, 2007; Witenberg, 2007; Bambulyaka, 2011; Brandt et al., 2015; Rapp and Freitag, 2015), despite evidence that they are related to yet distinct constructs (Van der Noll et al., 2010; Crawford, 2014; van Zalk and Kerr, 2014; Miklikowska, 2015). For instance, van Zalk and Kerr (2014) found that although intolerance and prejudice significantly reduced over time from early to late adolescence, there was a significant difference in their developmental trajectories. They also found that while a decline in intolerance was associated with the decline in prejudice, a lower level of prejudice was not associated with a lower level of intolerance (van Zalk and Kerr, 2014). Their findings highlighted two points: (a) Tolerance and prejudice are separate constructs with different causal pathways, and (b) tolerance and prejudice are inter-related but not equivalent constructs. As van Zalk and Kerr (2014) argued, tolerance and prejudice were only moderately correlated (i.e., *r* = −0.45), which indicate that only 20.25% (i.e., *r*^2^) or 45% (i.e., *r*) of the individual differences were shared between tolerance and prejudice (for a detailed discussion on the use of *r*^2^ or *r* as a percent of determination, please refer to Ozer, 1985). Despite the moderate correlation, the tolerance construct contained a unique variance that is not explained by prejudice.

Neuroscience research has also linked prejudice and tolerance to different neural structures (Amodio, 2014). For example, prejudice was strongly associated with the emotional centers of the brain (e.g., the amygdala, the orbital frontal cortex, and the insula; Beer et al., 2008; Chekroud et al., 2014), whereas tolerance is strongly associated with the goal-directed and behavioral regulation centers of the brain (e.g., the lateral prefrontal cortex, the anterior cingulate cortex, and the lateral prefrontal cortex; Bartholow et al., 2006; Beer et al., 2008; Amodio, 2014). These findings supported the argument that prejudice and tolerance should be treated as separate constructs. Conceptually, some researchers suggested treating prejudice and tolerance as two different forms of intergroup attitudes, with prejudice conceptualized as a preconceived negative evaluation of outgroup members (with historical, cultural, and developmental roots; Hjerm, 2005), while tolerance is conceptualized as a developmentally advanced moral reasoning ability coupled with prosocial beliefs and an understanding of equalitarian principles (e.g., social equality and equal rights; Miklikowska, 2015). Several researchers have argued for more systematic conceptual and empirical differentiation between prejudice and tolerance to understand how prejudice and tolerance interrelate (Butrus and Witenberg, 2013; van Doorn, 2014).

Tolerance consists of a cognitive component (i.e., awareness of difference and recognition of the problems of social discrimination and injustice), affective component (feelings of empathy and optimism), and behavioral component (willingness to act toward welcoming and integrating outgroup members; Cote and Erickson, 2009). Given that tolerance is linked with the capacity to hold multiple perspectives and accept differing values, it is no surprise that the disposition toward open-mindedness (i.e., openness factor) has been associated with tolerance (Weatherford and Spokane, 2013; Ackermann and Ackermann, 2015; Han and Pistole, 2017; Saef et al., 2019; Sparkman et al., 2019). In general, the openness factor has been positively linked with political tolerance (willingness to grant political rights to outgroups; Freitag and Rapp, 2015; Oskarsson and Widmalm, 2016), religious tolerance (willingness to recognize alternative religious faith; Proctor and McCord, 2009), universalism values (motivational goal toward social justice, equality, world peace, and unity with nature; Hamer et al., 2019), universal-diverse orientation (attitude of awareness and acceptance of both the similarities and differences among social groups; Han and Pistole, 2017), cross-cultural exploration (willingness to engage in activities aimed to further understand foreign cultures; Sturmer et al., 2013), multiculturalism (ideological belief in recognizing and appreciating ethnic differences in society; Sparkman et al., 2019), and multi-cultural competency (possesses cultural knowledge, awareness of the power dynamics among different cultural groups, and skillful in using culturally appropriate speech; Weatherford and Spokane, 2013).

Conversely, the openness factor has been negatively related to political conservatism (the ideological belief that opposes—any changes in social structure-, rejects uncertainty, and accepts status inequality Sibley et al., 2012), right-wing authoritarianism (adherence toward social norms and aggression toward others who challenged conventional norms; Leone et al., 2012; Sibley and Duckitt, 2013), dogmatism (unchangeable certainty about the truths of the beliefs of an individual and rejects all other beliefs; Batool and Akram, 2020), religious fundamentalism (a belief that there is only one true religion or one method of religious teaching, and views all other religions as evil or destructive; Proctor and McCord, 2009; Carlucci et al., 2011), and ethnocentrism (negative attitudes toward all groups other than the group of an individual; Hamer et al., 2019).

### The Present Research

As described in the preceding sections, the openness factor has been consistently linked with prejudice and tolerance (Ekehammar and Akrami, 2007; Sibley and Duckitt, 2008; Sturmer et al., 2013; Han and Pistole, 2017; Sparkman et al., 2019). However, the mechanism by which the openness factor protects against prejudice and promotes tolerance is still relatively unknown. In line with the recent evidence that prejudice and tolerance are separate constructs, this scoping review with meta-analysis examines how prejudice and tolerance relate to the various facets of the fundamental personality factor of openness to experience. To the best of our knowledge, there has been no attempt to review the extent of this literature and consolidate these findings into a coherent picture of the facet-level relationships between openness and the constructs of prejudice and tolerance. This scoping review supplemented with the meta-analytical synthesis of these facet-level relationships will respond directly to this need.

A scoping review is a particular type of systematic review that scopes for a potentially large and diverse body of literature relating to a research topic (Arksey and O'Malley, 2005). It is especially important for knowledge synthesis when there is a lack of understanding of key concepts within a topic (e.g., the lack of conceptual and empirical differentiation between prejudice and tolerance) and when a concept of interest is of a complex or heterogenous nature (e.g., the facet-level structure of openness; Pham et al., 2014). The scoping review offers a preliminary classification and systematization of the extant literature by providing a descriptive presentation of what is known about the key concepts, highlights the dominant methodologies used within the current literature, and identifies existing knowledge gaps (Peterson et al., 2017). Consequently, a scoping review was conducted on the existing literature on the openness facets and their association with prejudice and tolerance constructs. In addition, this review is supplemented with random-effect meta-analysis, which allowed us to reliably determine the average effect sizes of the associations between the facets of openness and the constructs of prejudice and tolerance from the available research in this area. Dependency of observations (i.e., the same group of experimenters or the same sample contributing to several effect sizes within the same model) was corrected as per the guidelines provided by Viechtbauer (2010). This scoping review with meta-analysis aims to enhance the existing knowledge in three ways: (a) providing the first review of the relationships between the facets of openness with prejudice and tolerance, (b) identifying the dominant measures of openness utilized in the existing literature, and (c) identifying possible research gaps in the literature to aid in the planning of future research on openness, prejudice, and tolerance.

## Methods

### Planning Stage

Based on the guidelines recommended by the Joanna Briggs Institute for a scoping review, the review objectives, selection criteria, and extraction methods for this review were planned, specified, and documented in a protocol. The protocol was preregistered and published (https://osf.io/yw9g8/) before data collection to provide transparency and limit the occurrence of reporting bias (Peters et al., 2020). The protocol specified that only studies examining the relationships between one or more facets of openness and prejudice or tolerance were included. Studies must investigate a personality construct that is explicitly positioned on the facet-level of the hierarchical model of personality (Figure 1) and measure personality as being a facet of the openness factor. Subject to this requirement, research studies examining all models and measures of openness, prejudice, and tolerance are eligible for inclusion.

There were no inclusionary or exclusionary criteria on participants or settings. That is, all studies that examined the relationships between the facets of openness and either prejudice or tolerance were included, regardless of the type of participants (e.g., university students and members of the general public) or the research context (e.g., geographic location and cultural setting). Only academic literature (i.e., journal articles, conference papers, dissertations, books, and book chapters) describing primary research was considered for inclusion in this review. This scoping review with meta-analysis considered experimental, quasi-experimental, and correlational study designs. Theoretical papers, reviews, and opinion papers were excluded. Finally, only articles that were published in English were included in the scoping review.

### Search Strategy

The scoping review utilized a three-step search strategy, as recommended by Peters et al. (2020). The first author conducted an initial search on Scopus and Web of Science between July and August 2020 using the following search terms: Openness AND facets AND Prejudice OR discrimination OR tolerance. From this initial search, key articles were identified, and the title, abstract, and keywords of these articles were screened for additional relevant search terms. An automation tool was also used to identify relevant search terms (Word Frequency Analyser; Clark et al., 2020). Following an iterative process, the authors finalized on the following search terms: (prejudice OR discrimination OR toleran^*^ OR intoleran^*^ OR diversity OR attitude^*^ OR religio^*^ OR ideology) for prejudice or tolerance, and [(openness OR intellect OR “big five” OR “five factor”) AND facet^*^] for openness facet(s).

**Figure 2:**
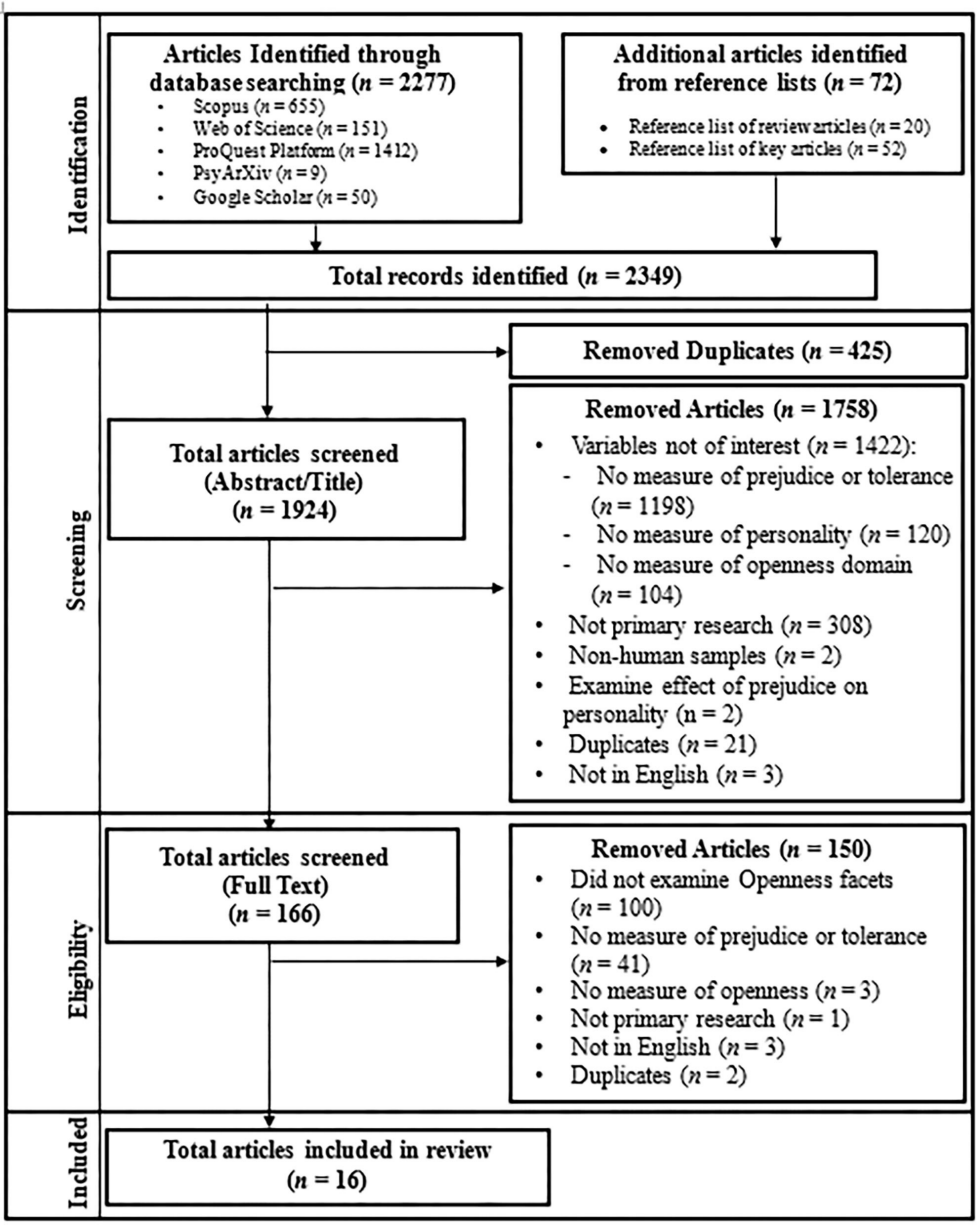
Screening and inclusion decision flowchart of scoping review with meta-analysis.

The main search using the finalized search terms was then conducted across three major databases (i.e., Scopus, Web of Science, and ProQuest), with the search string adapted to each database. A supplementary search of Google Scholar and PsyArXiv provided a further search of the relevant gray literature. A librarian liaison officer specialized in psychology was consulted and reviewed the search strategy in this stage. The last search examined the reference list of selected articles and relevant meta-analytic studies (e.g., Sibley and Duckitt, 2008; Crawford and Brandt, 2019) for articles relevant to the review questions. The reference list search identified an additional 72 articles. In total, 2,349 records were identified from our search strategy (for a detailed breakdown on the number of articles retrieved from each database, see Figure 2).

### Screening and Data Extraction Stage

Study selection involved screening all articles across two stages: (a) title and abstract screening, followed by (b) full-text screening (for a flowchart on the screening and selection process, see Figure 2). Citation files of all articles were first imported into a web-based systematic review software (Rayyan; Ouzzani et al., 2016) to aid the screening process. Screening tools were developed in advance (see Supplementary Materials), as per established guidelines (Polanin et al., 2019), to help reviewers evaluate the articles consistently and reliably. To assess for inter-reviewer reliability, all reviewers (i.e., authors of the current study) pilot-tested the screening tools on a sub-sample of 20 abstracts and attained a 90% inter-reviewer agreement, which satisfies the 75% minimum requirement (Tricco et al., 2018; Polanin et al., 2019). At least two reviewers screened all articles at each of the two screening stages. Articles that failed to meet the inclusionary criteria were excluded. Any disagreements that arose between the reviewers were resolved through discussion and achievement of consensus. The screening stage identified 16 articles that satisfied our review objectives and met all inclusion criteria (see Figure 2 for the complete PRISMA-ScR diagram; Tricco et al., 2018).

**Figure 3:**
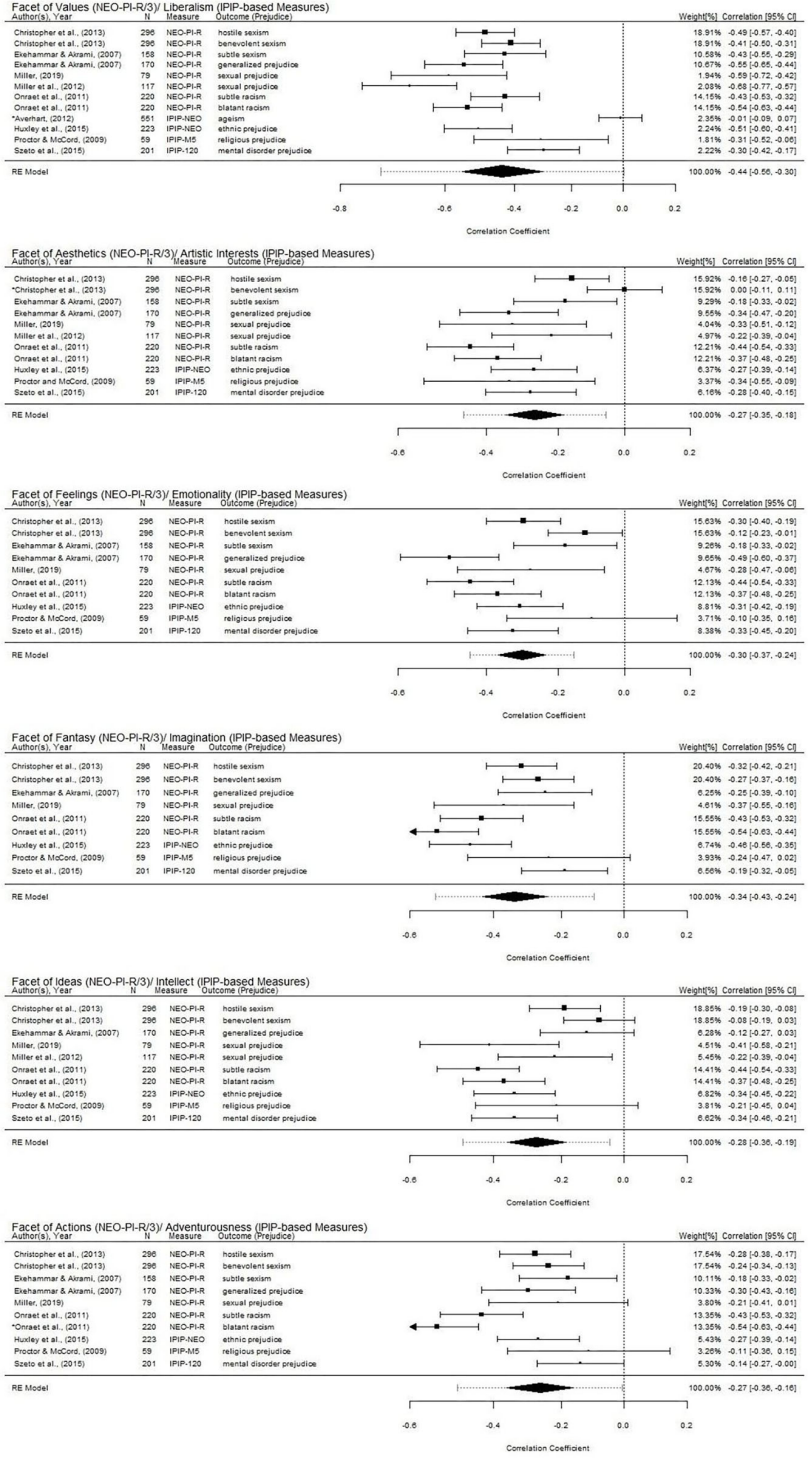
Forest plots for the associations of the facets of openness with prejudice. The summary effect sizes are displayed as a polygon at the bottom of each plot with the width reflecting the 95% confidence interval of the average effect size estimate. The dotted line extending from the polygon reflects the 95% prediction interval which accounts for both the uncertainty of the effect size estimate and the uncertainty in the between-study variance estimate (Riley et al., 2011). Studies with larger squares contributed more to the summary effect sizes compared to the other studies. Studies with an asterisk (*) were potential outliers and influential cases as per standardized residual, Cook's distances, and hat values for each model.

**Figure 4:**
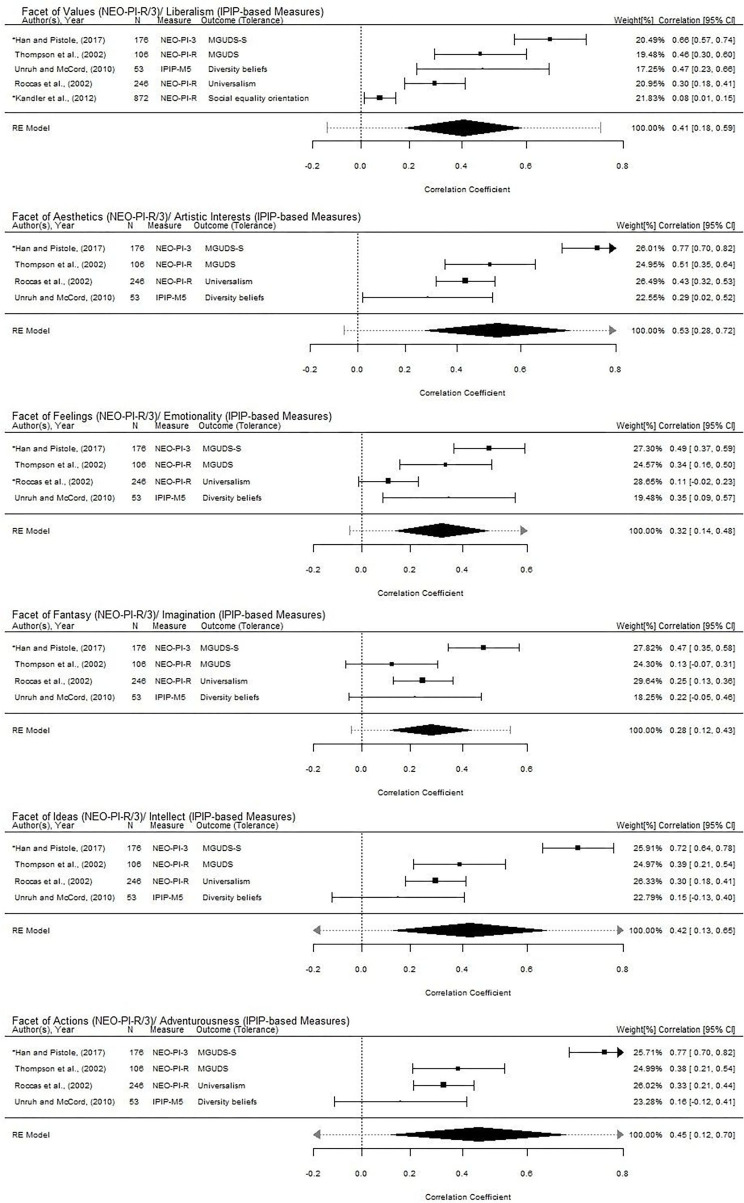
Forest plots for the associations of the facets of openness with tolerance. The summary effect sizes are displayed as a polygon at the bottom of each plot with the width reflecting the 95% confidence interval of the average effect size estimate. The dotted line extending from the polygon reflects the 95% prediction interval which accounts for both the uncertainty of the effect size estimate and the uncertainty in the between-study variance estimate (Riley et al., 2011). Studies with larger squares contributed more to the summary effect sizes compared to the other studies. Studies with an asterisk (*) were potential outliers and influential cases as per studentized deleted residuals, Cook's distances, and hat values for each model.

Using a preregistered data extraction tool (https://osf.io/yw9g8/), information pertinent to the review aims was extracted from the final set of 16 articles. The data extracted were as follows: author(s), year of publication, sample characteristics (sample size, age, gender, and sampling methods), country of research, personality measure used, type(s) of prejudice or tolerance examined, variables examined (i.e., name of the independent variables and dependent variables), measures used, and key findings. The data extraction tool was pilot tested on two articles by two reviewers (i.e., the first author and the last author of this paper). A high inter-reviewer agreement was achieved; there was no discrepancy in the information extracted from the two reviewers. The primary author extracted the remaining 14 articles. All extracted data were collated and stored using Microsoft Excel. The author(s), year of publication, participant demographics, country of research, personality measure used, outcome measure(s) used, and key findings are summarized in Table 2. All extracted results were categorized based on the relevance to the review aims and were summarized in Table 3. Where applicable, meta-analyses of the facet-level associations were conducted using the *metafor* (Viechtbauer, 2010) and *robumeta* (Fisher and Tipton, 2015) packages for R (R Development Core Team, 2015). Throughout our meta-analyses, we used random effects, with restricted maximum likelihood as the method for estimating random effects. In addition, because multiple effect sizes were sometimes taken from the same article (notably in Christopher et al., 2013), dependency was corrected for these effect sizes. That is, effect sizes taken from the same article were assigned to the same value of the random effect in the meta-analysis (for more details, see Konstantopoulos, 2011). Forest plots were created to present the summary effect sizes of the facet-level associations (Figures 3, 4). Two methods were used to assess for publication bias: (a) Egger's weighted regression method, which is suited for small sample meta-analyses (Egger et al., 1997), and (b) fail-safe *N*-test assessment among these meta-analyzed associations.

**Table 2:** Study characteristics, sample demographics, and measures used in the 16 included articles.

**Author(s)/Year**	**Study characteristics**				**Results**	
	***n, M*_**age**_, range**	**Country, sampling population**	**Personality measure used**	**Outcome measure(s) used**	**Association of openness facets with indices of prejudice**	**Association of openness facets with indices of tolerance**
**A**						
1. Anglim et al. (2017)	*n* = 1,244 (47% female), *M*_age_ = 44.3, range = 18–70	Australia, community sample	HEXACO-PI (Lee and Ashton, 2004)	57-item Portrait Values Questionnaire (PVQ; Schwartz et al., 2012)	Not investigated	• Aesthetic appreciation • Universalism (*r* = 0.39^***^) • Inquisitiveness • Universalism (*r* = 0.31^***^) • Creativity • Universalism (*r* = 0.23^***^) • Unconventionality • Universalism (*r* = 0.36^***^)
2. Anglim et al. (2019)	*n* = 731 (66% female), *M*_age_ = 43.0, *sd* = 12.0	Australia, community sample	HEXACO-PI (Lee and Ashton, 2004)	• A 16-item measure was developed to assess four types of prejudice • Attitudes Toward Diversity Scale (Montei et al., 1996)	• Aesthetic appreciation • Sexism (*r* = −0.12^**^), racism (*r* = −0.12^**^), ageism (*r* = −0.12^**^), disability prejudice (*r* = −0.17^***^) • Inquisitiveness • Sexism (*r* = −0.06, n.s), racism (*r* = −0.11^**^), ageism (*r* = −0.09^*;^), disability prejudice (*r* = −0.10^**^) • Creativity • Sexism (*r* = −0.06, n.s), racism (*r* = −0.07, n.s), ageism (*r* = −0.11^**^), disability prejudice (*r* = −0.11^**^) • Unconventionality • Sexism (*r* = −0.05, n.s), racism (*r* = −0.08^*;^), ageism (*r* = −0.05, n.s), disability prejudice (*r* = −0.05, n.s)	• Aesthetic appreciation • Diversity attitude (*r* = 0.19^***^) • Inquisitiveness • Diversity attitude (*r* = 0.18^***^) • Creativity • Diversity attitude (*r* = 0.13^***^) • Unconventionality • Diversity attitude (*r* = 0.16^***^)
3. Averhart (2012)	*n* = 551 (55% female), *M*_age_ = 40.58, range = 23–71	United States, community sample	IPIP-NEO (Goldberg, 1999)	29-item Fraboni Scale of Ageism (Fraboni et al., 1990)	• Liberalism • Ageism (*r* = −0.011, n.s.)	Not investigated
4. Christopher et al. (2013)	*n* = 296 (48% female), *M*_age_ = 39.81, range = 22–74	United States, community sample	NEO-PI-R (Costa and McCrae, 1992)	22-item Ambivalent Sexism Inventory (Glick and Fiske, 1996)	• Values • Sexism (hostile, *r* = −0.49^**^; benevolent, *r* = −0.41^**^) • Aesthetics • Sexism (hostile, *r* = −0.16^**^; benevolent, *r* = 0.00, n.s) • Feelings • Sexism (hostile, *r* = −0.30^**^; benevolent, *r* = −0.12, n.s) • Fantasy • Sexism (hostile, *r* = −0.32^**^; benevolent, *r* = −0.27^**^) • Ideas • Sexism (hostile, *r* = −0.19^**^; benevolent, *r* = −0.08, n.s) • Actions • Sexism (hostile, *r* = −0.28^**^; benevolent, *r* = −0.24^**^)	Not investigated
**B**						
5a. Ekehammar and Akrami (2007) (Study 1)	*n* = 158 (50% female), *M*_age_ = 24.7, range = 19–50	Sweden, college students and community sample	NEO-PI-R (Costa and McCrae, 1992)	8-item Swedish Modern Sexism Scale (Ekehammar et al., 2000)	• Values (subtle sexism, *r* = −0.43^*^) • Aesthetics(subtle sexism, *r* = −0.18^*^) • Feelings (subtle sexism, *r* = −0.18^*^) • Fantasy (subtle sexism, *r* = not disclosed) • Ideas (subtle sexism, *r* = not disclosed) • Actions (subtle sexism, *r* = −0.18^*^)	Not investigated
5b. Ekehammar and Akrami (2007) (Study 2)	*n* = 170 (63% female), *M*_age_ = 19.9, range = 16–50	Sweden, college and high school students	NEO-PI-R (Costa and McCrae, 1992)	• 9-item Modern Racial Prejudice Scale (Akrami et al., 2000) • 8-item Swedish Modern Sexism Scale (Ekehammar et al., 2000) • 11-item Modern Attitude Toward People with Mental Disabilities Scale (Akrami et al., 2006) • 10-item Attitude to Homosexuality Scale (Akrami et al., 2006)	• Values (generalized prejudice, *r* = −0.55^*^) • Aesthetics (generalized prejudice, *r* = −0.34^*^) • Feelings (generalized prejudice, *r* = −0.49^*^) • Fantasy (generalized prejudice, *r* = −0.25^*^) • Ideas (generalized prejudice, *r* = −0.12, n.s.) • Actions (generalized prejudice, *r* = −0.30^*^)	Not investigated
6. Han and Pistole (2017)	*n* = 176 (72% female), *M*_age_ = 21.01, range = 18–51	United States, college students	NEO-PI-3 (McCrae et al., 2005)	15-item Miville-Guzman Universal-Diverse Scale-Short Form (Fuertes et al., 2000)	Not investigated	• Values (UDO, *r* = −0.66^*^) • Aesthetics (UDO, *r* = −0.77^*^) • Feelings (UDO, *r* = −0.49^*^) • Fantasy (UDO, *r* = −0.47^*^) • Ideas (UDO, *r* = −0.72^*^) • Actions (UDO, *r* = −0.77^*^)
7. Huxley et al. (2015)	*n* = 223 (59% female), *M*_age_ = 29.83, *sd* = 13.59	Australia, college students and community sample	IPIP-NEO (Goldberg, 1999)	6-item feeling thermometer scale on attitudes toward asylum seeker ethnic groups (i.e., Sri Lankans, Afghanis, Iraqis, Sudanese, Burmese, and asylum seekers in general)	• Liberalism (ethnic prejudice, *r* = −0.51^**^) • Artistic interests (ethnic prejudice, *r* = −0.27^**^) • Emotionality (ethnic prejudice, *r* = −0.31^**^) • Imagination (ethnic prejudice, *r* = −0.46^**^) • Intellect(ethnic prejudice, *r* = −0.34^**^) • Adventurousness (ethnic prejudice, *r* = −0.27^**^)	Not investigated
**C**						
8. Kandler et al. (2012)	*n* = 872 (74% female), *M*_age_ = 34.3, range = 17–82	Germany, community sample	NEO-PI-R (Costa and McCrae, 1992)	Eight bipolar items were developed and used to examine orientation toward equality	Not investigated	• Values (social equality orientation, *r* = −0.08^*^)
9. Miller (2019)	*n* = 79 (54% female), *M*_age_ = 22.08, range = 18–39	United States, college students	NEO-PI-R (Costa and McCrae, 1992)	Two items from the Right-Wing Authoritarianism (RWA) scale were used to examine homosexuality and “different” sexual preference	• Values (sexual prejudice, *r* = −0.59^***^) • Aesthetics (sexual prejudice, *r* = −0.33^**^) • Feelings (sexual prejudice, *r* = −0.28^*^) • Fantasy (sexual prejudice, *r* = −0.37^**^) • Ideas (sexual prejudice, *r* = −0.41^***^) • Actions (sexual prejudice, *r* = −0.21, n.s)	Not investigated
10. Miller et al. (2012)	*n* = 117 (89% female), *M*_age_ = 20.69, *sd* = 4.41	United States, college students	NEO-PI-R (Costa and McCrae, 1992)	• 20-item Attitudes Toward Lesbians and Gay Men scale (Herek, 1988) • 10-item Attitudes Toward Homosexuals scale (Agnew et al., 1993)	• Values (sexual prejudice, *r* = −0.68^***^) • Aesthetics (sexual prejudice, *r* = −0.22^*^) • Feelings (sexual prejudice, *r* = not disclosed) • Fantasy (sexual prejudice, *r* = not disclosed) • Ideas (sexual prejudice, *r* = −0.22^*^) • Actions (sexual prejudice, *r* = not disclosed)	Not investigated
11. Onraet et al. (2011)	*n* = 220 (50% female), *M*_age_ = 46, range = 17–86	Belgium, community sample	NEO-PI-R (Costa and McCrae, 1992)	• 9-item blatant racism scale (Duriez and Van Hiel, 2002) • 12-item subtle racism scale (Van Hiel and Mervielde, 1996)	• Values • Racism (blatant, *r* = −0.54^***^; subtle, *r* = −0.43^***^) • Aesthetics • Racism (blatant, *r* = −0.44^***^; subtle, *r* = −0.37^***^) • Feelings • Racism (blatant, *r* = −0.44^***^; subtle, *r* = −0.37^***^) • Fantasy • Racism (blatant, *r* = −0.54^***^; subtle, *r* = −0.43^***^) • Ideas • Racism (blatant, *r* = −0.44^***^; subtle, *r* = −0.37^***^) • Actions • Racism (blatant, *r* = −0.54^***^; subtle, *r* = −0.43^***^)	Not investigated
12. Proctor and McCord (2009)	*n* = 59 (na), *M*_age_ = not available, range = not available	United States, college students	IPIP-M5 (McCord, 2002)	Four-item measure was developed and used to examine prejudice toward Muslim	• Liberalism (religious prejudice, *r* = −0.309^*^) • Artistic interests (religious prejudice, *r* = −0.339^**^) • Emotionality (religious prejudice, *r* = −0.1, n.s) • Imagination (religious prejudice, *r* = −0.238, n.s) • Intellect (religious prejudice, *r* = −0.214, n.s) • Adventurousness (religious prejudice, *r* = −0.114, n.s)	Not investigated
**D**						
13. Roccas et al. (2002)	*n* = 246 (65% female), *M*_age_ = 22, range = 16–35	Israel, college students	NEO-PI-R (Costa and McCrae, 1992)	62-item (Schwartz, 1992) value inventory	Not investigated	• Values (universalism value, *r* = 0.30^**^) • Aesthetics (universalism value, *r* = 0.43^**^) • Feelings (universalism value, *r* = 0.11^**^) • Fantasy (universalism value, *r* = 0.25^**^) • Ideas(universalism value, *r* = 0.30^**^) • Actions (universalism value, *r* = 0.33^**^)
14. Szeto et al. (2015)	*n* = 201 (71% female), *M*_age_ = 20.52, *sd* = 3.57	Canada, college students	IPIP-120 (not disclosed in the study)	• A 27-item was developed and used to assess prejudice toward people with mental disorders • 12-item social distance questionnaire (Norman et al., 2008)	• Liberalism (mental disorder prejudice, *r* = −0.30^**^) • Artistic interests (mental disorder prejudice, *r* = −0.28^**^) • Emotionality (mental disorder prejudice, *r* = −0.33^**^) • Imagination (mental disorder prejudice, *r* = −0.19^**^) • Intellect (mental disorder prejudice, *r* = −0.34^**^) • Adventurousness (mental disorder prejudice, *r* = −0.14, n.s)	Not investigated
15. Thompson et al. (2002)	*n* = 106 (86% female), *M*_age_ = 34.7, range = 22–57	United States, college students	NEO-PI-R (Costa and McCrae, 1992)	45-item Miville-Guzman Universality-Diversity Scale (Miville et al., 1999)	Not investigated	• Values (UDO, *r* = 0.46^**^) • Aesthetics (UDO, *r* = 0.51^**^) • Feelings (UDO, *r* = 0.34^**^) • Fantasy (UDO, *r* = 0.13, n.s) • Ideas (UDO, *r* = 0.39^**^) • Actions (UDO, *r* = 0.38^**^)
16. Unruh and McCord (2010)	*n* = 53 (72% female), *M*_age_ = 20.94, range = 18–37	United States, college students	IPIP-M5 (McCord, 2002)	25-item Professional Beliefs About Diversity Scale (Pohan and Aguilar, 2001)	Not investigated	• Liberalism (diversity belief, *r* = 0.47^**^) • Artistic interests (diversity belief, *r* = 0.29^*^) • Emotionality (diversity belief, *r* = 0.35^*^) • Imagination (diversity belief, *r* = 0.22, n.s) • Intellect (diversity belief, *r* = 0.15, n.s) • Adventurousness (diversity belief, *r* = 0.16, n.s)

*** *p < 0.001*,

** *p < 0.01, and*

* *p < 0.05. n.s. refers to non-significance finding*.

*The effect sizes in Christopher et al. (2013) were corrected for dependency*.

*The effect sizes of the two studies in Ekehammar and Akrami (2007) corrected for dependency*.

*The effect sizes in Onraet et al. (2011) article were corrected for dependency*.

**Table 3:** Categorization of included studies.

**Types of personality measure**	**No of studies (i.e., *k*)**	**Types of prejudice and tolerance measured**
NEO-PI-R/3	10	*Prejudice (k = 6)*
		Sexism (4, 5a)
		Generalized prejudice (5b)
		Sexual prejudice (9, 10)
		Racism (11)
		*Tolerance (k = 4)*
		Universal-Diverse Orientation (6, 15)
		Social equality orientation (8)
		Universalism value (13)
IPIP-based measure	5	*Prejudice (k = 4)*
		Ageism (3)
		Ethnic prejudice (7)
		Religious prejudice (12)
		Mental disorder prejudice (14)
		*Tolerance (k = 1)*
		Diversity beliefs (16)
HEXACO-PI	2^a^	*Prejudice(k = 1)*
		Sexism, Racism, Ageism, and Disability prejudice (2)
		*Tolerance (k = 2)*
		Diversity attitude (2)
		Universalism value (1)

*The number reflected in the parentheses refers to the articles outlined in Table 2*.

a *As there are only two unique studies that used HEXACO-PI, the total number of studies for HEXACO-PI is indicated as two in this table*.

## Results

### Study Demographics and Characteristics

Following the Joanna Briggs Institute framework for scoping review (Peters et al., 2020) and the inclusion criteria outlined above, a total of 16 articles were identified (Figure 2). That is, 14 peer-reviewed publications, one book chapter (Huxley et al., 2015), and one PhD dissertation (Averhart, 2012) were identified to be relevant to the review objective (Table 2). The 16 articles contributed 17 separate studies (Table 3) that investigated the facet level associations of openness and indices of prejudice or tolerance.

All 17 studies were published between 2002 and 2019, most published in the last 10 years (i.e., between 2012 and 2021; *k* = 11, 65%). With regard to geographic and cultural representation among these articles, they were from the United States (*k* = 8, 47%), Australia (*k* = 3, 17%), Belgium (*k* = 1, 6%), Canada (*k* = 1, 6%), Germany (*k* = 1, 6%), and Sweden (*k* = 2, 12%). The only research study from a non-Western setting was conducted in Israel (*k* = 1, 6%). No studies from Africa, South America, and other major parts of Asia (i.e., central, south, east, or south-east Asia) were identified in this review. All 17 studies used convenience sampling methods (Table 2).

Table 3 shows the categorization of studies based on the openness measures used and the indices of prejudice or tolerance examined. The most frequently used measure of facets of openness was the revised neuroticism-extraversion-openness personality inventory (NEO-PI-R) and NEO-PI-3, collectively labeled as NEO-PI-R/3 (*k* = 10, 59%; Table 3). Other measures of facets of openness were IPIP-based measures (*k* = 5, 29%) and the HEXACO-PI (*k* = 2, 12%). The dominant use of the NEO-PI-R/3 was found in both tolerance and prejudice studies (Table 3). Four of the seven articles (57%) that examined tolerance used the NEO-PI-R/3 to assess the facets of openness, and 6 of the 11 articles (50%) that examined prejudice used the NEO-PI-R/3 to assess the facets of openness (Table 3).

### Facets of Openness and Prejudice

Eleven studies examined prejudice as the dependent variable (Table 3). All 11 studies used self-report questionnaires to measure the indices of prejudice; none of the studies include implicit or behavioral measures of prejudice (Table 2). Out of the 11 studies, nine studies used established scales to assess prejudice, while two studies developed their prejudice measures (i.e., Proctor and McCord, 2009; Anglim et al., 2019). Together, these 11 studies examined a total of nine different types of prejudice (Table 3), namely: ageism (*k* = 2), sexual prejudice (*k* = 2), sexism (*k* = 3), racism (*k* = 2), ethnic prejudice (*k* = 1), mental disorder prejudice (*k* = 1), religious prejudice (*k* = 1), disability prejudice (*k* = 1), and generalized prejudice (*k* = 1). All studies used different measures to assess prejudice; no two studies used the same prejudice measure (Table 2).

Among studies that examined prejudice and used the NEO-PI-R/3 (*k* = 6; Table 3), the openness facet of values was consistently linked with all types of prejudice examined (i.e., sexism, generalized prejudice, sexual prejudice, and racism; Table 2 and Figure 3). Among studies that examined prejudice and used international personality item pool (IPIP)-based measures (*k* = 4), the facets of liberalism and artistic interests were associated with most indices of prejudice (Table 2 and Figure 3). Only one study examined prejudice and used the honesty-humility, emotionality, extraversion, agreeableness, and conscientiousness and openness to experience personality inventory (HEXACO-PI), and of the four indices of prejudice examined (i.e., sexism, racism, ageism, and disability prejudice), only the facet of aesthetic appreciation was associated with all indices of prejudice (Table 2).

### Facets of Openness and Tolerance

Seven studies examined tolerance as the dependent variable (Table 3). All seven studies used self-report questionnaires to measure tolerance, and none of the studies included any implicit or behavioral measure (Table 2). Most of the studies used established scales to assess tolerance, except for Kandler et al. (2012), who developed their tolerance measure. The seven studies examined five different types of tolerance, namely, universal-diverse orientation (UDO; *k* = 2), social equality orientation (*k* = 1), universalism value (*k* = 2), diversity beliefs (*k* = 1), and diversity attitudes (*k* = 1; Table 3).

Among the studies that examined tolerance and used the NEO-PI-R/3 (*k* = 4; Table 3), the openness facet of values was associated with all indices of tolerance (i.e., universal-diverse orientation, universalism value, and social equality orientation; Table 2 and Figure 4). The study that used IPIP-based measure found only the openness facets of liberalism, artistic interests, and emotionality to be associated with diversity beliefs (Table 2 and Figure 4). Studies that examined tolerance and used the HEXACO-PI (*k* = 2) found all openness facets within HEXACO-PI (i.e., aesthetic appreciation, inquisitiveness, creativity, and unconventionality) to be associated with tolerance (i.e., diversity attitude and universalism value; Table 2).

### Overall Effect Size (*r*) Aggregated Across Studies

Following the procedures outlined by Quintana (2015), we conducted a series of random effect meta-analyses on the associations of the facets of openness in NEO-PI-R and IPIP-based measures (see Figures 3, 4 for the forest plots). HEXACO facets of openness were excluded from this analytic strategy due to the limited number of studies. In addition, as the facets of openness in the IPIP-based measures (i.e., liberalism, artistic interests, emotionality, imagination, intellect, and adventurousness; Goldberg, 1999) were developed as proxy measures of the facets of openness in NEO-PI-R (i.e., values, aesthetics, feelings, fantasy, ideas, and actions), the facets of openness that were similar in theme were categorized together in the meta-analyses (see Table 1 for the detailed description of the theme and definition of the facets of openness included in the meta-analyses). The aggregated correlation estimates with prejudice for the facets of openness were as follows: values/liberalism (*r* = −0.44, 95% CI: −0.56, −0.30), aesthetics/artistic interests (*r* = −0.27, 95% CI: −0.35, −0.18), feelings/emotionality (*r* = −0.30, 95% CI: −0.37, −0.24), fantasy/imagination (*r* = −0.34, 95% CI: −0.43, −0.24), ideas/intellect (*r* = −0.28, 95% CI: −0.36, −0.19), and actions/adventurousness (*r* = −0.27, 95% CI: −0.36, −0.16; see Figure 3). Publication bias was assessed using Egger's (1997) regression test and Rosenthal's (1979) fail-safe *N*-test. Egger's regression tests of these associations were not significant, indicating a lack of bias (values/liberalism: *z* = −0.72, *p* = 0.47; aesthetics/artistic interests: *z* = −0.69, *p* = 0.49; feelings/emotionality: *z* = 1.18, *p* = 0.24; fantasy/imagination: *z* = 0.49, *p* =0.62; ideas/intellect: *z* = −0.13, *p* = 0.89; actions/adventurousness: *z* = 1.12, *p* = 0.26). According to Rosenthal's (1979) fail-safe *N*-test, a value that is five times greater than the number of studies included in the meta-analyses is needed to indicate a lack of publication bias (i.e., 60). The values of fail-safe *N*-test were 2,131 (values/liberalism), 536 (aesthetics/artistic interests), 645 (feelings/emotionality), 729 (fantasy/imagination), 495 (ideas/intellect), and 578 (actions/adventurousness) that all exceeded the criterion.

With regard to the aggregated correlation estimates of the facets of openness with tolerance, our results were as follows: values/liberalism (*r* = 0.41, 95% CI: 0.18, 0.59), aesthetics/artistic interests (*r* = 0.53, 95% CI: 0.28, 0.72), feelings/emotionality (*r* = 0.32, 95% CI: 0.14, 0.48), fantasy/imagination (*r* = 0.28, 95% CI: 0.12, 0.43), ideas/intellect (*r* = 0.42, 95% CI: 0.13, 0.65), and actions/adventurousness (*r* = 0.45, 95% CI: 0.12, 0.70; Figure 4). There were no indications of publication bias according to Egger's regression test and Rosenthal's fail-safe *N*-test. Egger's regression tests of these associations did not yield significant results (values/liberalism: *z* = 1.16, *p* = 0.25; aesthetics/artistic interests: *z* = −0.89, *p* = 0.38; feelings/emotionality: *z* = 0.39, *p* = 0.69; fantasy/imagination: *z* = −0.59, *p* = 0.55; ideas/intellect: *z* = −0.87, *p* = 0.39; actions/adventurousness: *z* = −0.89, *p* = 0.37). Given the criterion of exceeding - the value of 25 (i.e., five times greater than the number of studies included in the meta-analyses),- the fail-safe *N*-test results of 251 (values/liberalism), 295 (aesthetics/artistic interests), 79 (feelings/emotionality), 64 (fantasy/imagination), 175 (ideas/intellect), and 210 (actions/adventurousness) provided no evidence of publication bias.

As our meta-analyses had shown, the facets of openness included in the NEO-PI-R/3 and its equivalent IPIP-based measures were all significantly associated with prejudice and tolerance. That is, the aggregated effect sizes of the relationship between each of these facets with prejudice and tolerance were all statistically significant (Figures 3, 4). Among the facets of openness in both the NEO-PI-R/3 and its equivalent IPIP-based measures, the facets of values (or liberalism), feelings (or emotionality), and fantasy (or imagination) were consistently linked with both prejudice (values/liberalism: *r* = −0.44; feelings/emotionality: *r* = −0.30; fantasy/imagination: *r* = −0.34) and tolerance (values/liberalism: *r* = 0.53; feelings/emotionality: *r* = 0.32; fantasy/imagination: *r* = 0.28). Our review also found specific facets of openness to be associated more strongly with indices of tolerance than with indices of prejudice. For instance, the aggregated correlation estimates found the facets of aesthetics (or artistic interests), ideas (or intellect), and actions (or adventurousness) were associated more strongly with tolerance (aesthetics/artistic interests: *r* = 0.53; ideas/intellect: *r* = 0.42; actions/adventurousness: *r* = 0.45) than prejudice (aesthetics/artistic interests: *r* = −0.27; ideas/intellect: *r* = −0.28; actions/adventurousness: *r* = −0.27).

Heterogeneity of the facet-level effect sizes was assessed using the *Q* and *I*^2^ statistics. All Q statistics reached statistical significance, meaning that there is significant heterogeneity in the effect sizes of our included studies. In other words, the facet-level associations of openness with prejudice and tolerance do not share common effect sizes (Table 4). The *I*^2^ values, which indicate the percentage of total variation due to heterogeneity, ranged from 48.90 to 94.45%, meaning that most of the variations observed in the associations were largely due to between-study differences (Higgins et al., 2003). There are methods that could help identify the sources of heterogeneity in a meta-analysis (e.g., subgroup analysis and meta-regression; Song et al., 2001). However, due to the limited number of studies, these analyses were not conducted in this review. Sensitivity analyses (i.e., standardized residual, studentized deleted residuals, Cook's distances, and hat values; Viechtbauer and Cheung, 2010) also revealed the presence of outliers and influential studies. In sum, the large prediction interval of these facet-level associations (i.e., 95% prediction intervals of the associations with tolerance were all involved zero), the presence of outliers and influential cases, and the limited number of studies suggest that caution are warranted in the generalization of these aggregated correlation estimates.

**Table 4:** Heterogeneity statistics for the associations between the facets of openness and the two outcome variables (prejudice and tolerance).

**Outcomes and facets**	***k***	**Between-group effect (*Q*)**	**% of total variance due to heterogeneity (*I*^**2**^)**	**95% CI for** ***I***^**2**^	
				**LL**	**UL**
**Prejudice**					
Values/Liberalism	12	141.51^***^	91.37	79.98	97.34
Aesthetics/Artistic Interests	11	39.74^***^	65.09	32.82	88.97
Feelings/Emotionality	10	31.32^***^	48.90	5.15	86.57
Fantasy/Imagination	9	29.57^***^	72.74	35.76	93.09
Ideas/Intellect	10	33.39^***^	68.99	34.16	91.01
Actions/Adventurousness	10	36.30^***^	74.25	40.87	93.38
**Tolerance**					
Values/Liberalism	5	86.78^***^	93.63	81.82	99.18
Aesthetics/Artistic Interests	4	39.31^***^	91.85	73.93	99.42
Feelings/Emotionality	4	18.84^***^	79.38	39.08	98.20
Fantasy/Imagination	4	11.52^**^	73.47	16.61	97.94
Ideas/Intellect	4	44.46^***^	92.71	76.75	99.48
Actions/Adventurousness	4	58.40^***^	94.45	82.36	99.60

*** *p < 0.001 and*

** *p < 0.01*.

## Discussion

The objectives of this review were to (a) comprehensively map the extant research on the relationships between facets of openness and prejudice/tolerance, (b) identify which facets have been most consistently linked with prejudice and tolerance, and (c) to further characterize the literature in terms of the specific openness measures used. To fulfill these objectives, this scoping review with meta-analysis adopted the systematic approach recommended by the Joanna Briggs Institute (Peters et al., 2020) and identified 16 relevant research articles. In this section, we first provide an overall summary of the review findings, followed by a discussion on the theoretical implications of specific findings. Lastly, we offer directions for future research and to note the limitations of this review.

### Forms of Prejudice and Tolerance Represented in the Literature

The studies included in this review examined nine different forms of prejudice: ageism, racism, ethnic prejudice, sexual prejudice, sexism, mental disorder prejudice, religious prejudice, disability prejudice, and generalized prejudice (i.e., an aggregation of various forms of target-specific prejudice; Table 3). While these nine forms represent a reasonably broad cross-section of prejudice and discrimination literature (Duckitt, 1992; Sibley and Duckitt, 2008; Son Hing and Zanna, 2010), several prominent forms of prejudice (e.g., gender identity prejudice and anti-immigrant prejudice) have not yet been examined in terms of their relationships with openness facets. In addition, not all measures of prejudice were reviewed in our included studies. For example, none of our studies include an implicit measure of prejudice (e.g., the Implicit Association Test; IAT; Greenwald et al., 1998). Using implicit means to examine prejudice had been found to produce stronger indices of prejudice than those using self-report measures (e.g., Legault et al., 2007; Nosek et al., 2007). Some researchers regarded implicit measure of prejudice to be a more reflective measure of prejudice as participants are less able to control their responses to the measure and, hence, less likely to respond in a socially desirable manner (e.g., Cvencek et al., 2010; Geoffrey, 2013). Several studies had found participants who were motivated to suppress their prejudice (to avoid negative reactions from others) were more likely to report a lower prejudice score on self-report measures than implicit measures of prejudice (e.g., Devine et al., 2002; Legault et al., 2007). Therefore, it is likely that our included studies, which used only self-report measures of prejudice, captured only a thwarted level of prejudice, especially among participants who are motivated to suppress their prejudice for social desirability.

Our review identified five different operationalization of the tolerance construct, i.e., universal-diverse orientation, social equality orientation, universalism value, diversity beliefs, and diversity attitudes (Table 3). It is noteworthy that research into the psychology of tolerance began only recently in the 1990s in response to the growing tension between immigrants and the local citizens (Plaut, 2010). The relatively new field also meant that the characterization of the construct is still ongoing, and researchers did not consistently agree upon its operational definition. For instance, the definition for tolerance had ranged from (a) enduring things that one disliked or disapproved without interference (Van der Walt, 2014), (b) having a positive orientation toward minorities (Cote and Erickson, 2009), (c) adopting Egalitarian political ideology and attitude (Morley, 2003; Vasiljevic and Crisp, 2013; van Zalk and Kerr, 2014), (d) putting up with others for social harmony (Gibson, 1992; Vogt, 1994), and (e) embracing diverse ideas and opposing values (Freitag and Rapp, 2015). We argued that the lack of a clear and agreed-upon definition of tolerance is the key reason researchers have been unable to differentiate tolerance from prejudice. As found by our review, current literature operationalized the tolerance construct as either a (a) positive orientation toward differences (i.e., universal-diverse orientation, diversity beliefs, and diversity attitudes), (b) Egalitarian ideology (i.e., social equality orientation), or (c) universalism value (i.e., the pursuit of welfare and protection for all individuals). Several researchers have argued that Egalitarianism is not sufficient in promoting a truly tolerant society (Son Hing et al., 2008). Instead, researchers have advocated for tolerance to be operationalized as a positive orientation toward diversity, which is characterized by the awareness of intergroup differences, appreciation of diversity, and having a sense of relatedness toward outgroup members (Miville et al., 1999; Son Hing et al., 2008; Hjerm et al., 2019). As only seven studies on tolerance were retrieved, and only four studies operationalized tolerance as a positive orientation toward diversity (Table 3), it is recommended for future research to explore the operationalization of tolerance as an orientation toward diversity. More primary research studies on the association between the facets of openness and tolerance are also needed to broaden our understanding of the personality underpinning of this important aspect of tolerance.

### Openness Facets and Their Relationships With Prejudice and Tolerance

Our review aimed to examine whether the relationships between the facets of openness and indices of prejudice and tolerance differ depending on how the openness facets were measured. Logically, this has to be the case since disagreement and uncertainty over the nature and the structure of openness (de Raad and van Heck, 1994; Hough and Ones, 2001; Woo et al., 2014a; Christensen et al., 2019) have resulted in different measures aligned with different theoretical perspectives. However, the small number of studies limited our ability to make systematized comparisons between personality measures. For instance, only one study examined prejudice using honesty-humility, emotionality, extraversion, agreeableness, conscientiousness, and openness to experience personality inventory (HEXACO-PI), and only one study examined tolerance using IPIP-based measures.

Within the identified studies, the NEO-PI-R and its variants were identified as the most frequently used measures of openness facets (Table 3). Several studies have found that the NEO-PI-R/3 facets of openness mainly characterize the individual differences in openness toward non-intellectual experiences, such as aesthetic experiences, variety-seeking, daydreaming, and emotions (Table 1; e.g., Woo et al., 2014a; Christensen et al., 2019). While the NEO-PI-R/3 provides some coverage of individual differences in openness toward intellectual pursuits, such as intellectual curiosity toward ideas (i.e., the facet of ideas), several facets associated with intellectual pursuit are not captured (e.g., ingenuity, scientific curiosity, depth, and self-assessed intelligence; Woo et al., 2014a; Christensen et al., 2019). Therefore, reliance on the NEO-PI-R/3 represents a weakness of the extant literature, since investigations of facet-level relationships between openness and prejudice do not examine the full breadth of the openness construct.

In our meta-analyses, we found the facets of values, feelings, and fantasy to be consistently linked with both prejudice and tolerance. The facets of feelings and fantasy represent the sensitivity and receptiveness of an individual toward inner processes (Albrecht et al., 2014). That is, individuals who scored high in the facet of feelings experience more differentiated emotional states (e.g., feeling the anxiety of meeting unfamiliar others while appreciating the excitement of meeting someone new), and individuals who scored high in the facet of fantasy are inclined toward creating a pleasant experiential and inner world while rejecting social norms and social expectations (Figure 1; Costa and McCrae, 1992; Connelly et al., 2014). These attributes of the facets of feelings and fantasy may explain how individuals high in these two facets could better appreciate differences (i.e., high in tolerance) while holding a pleasant attitude of diverse others (i.e., low in prejudice). The facet of values, which refers to the willingness of an individual to reexamine social, political, cultural, and religious values (Costa and McCrae, 1992), has also been positively linked with dispositional perspective taking (Miller, 2019) and negatively linked with right-wing authoritarianism (Sibley and Duckitt, 2010). Therefore, it may be argued that the facets of values, feelings, and fantasy promote tolerance and protect an individual against prejudiced attitudes via enhancing the ability to adopt the perspectives of others, resisting various forms of dogmatic and authoritarian attitudes, and increasing the motivation to shape pleasant intergroup relationships.

Our meta-analyses also found the facets of aesthetics, ideas, and actions to be linked more strongly with tolerance than prejudice. These facets (aesthetics, ideas, and actions) represent the sensitivity and receptiveness of an individual toward the external environment (Griffin and Hesketh, 2004). For instance, the individuals high in the facet of aesthetics are attuned toward appreciating beauty in their environment (e.g., natural, physical, and social environment), the individuals high in the facet of ideas are attuned to intellectual concepts (e.g., beliefs, worldviews, and philosophy), and individuals high in the facet of actions actively sought out new and unusual experiences (e.g., trying foreign foods, working in foreign countries, and traveling to exotic countries; Albrecht et al., 2014). The sensitivity toward the external environment and the receptiveness toward diverse experiences might influence tolerance more than prejudice. Similarly, the HEXACO-PI facet of unconventionality (i.e., the willingness to accept the new and unusual) were moderately associated with tolerance (universalism: *r* = 0.36, *p* < 0.001; diversity attitude: *r* = 0.16, *p* < 0.001) but weakly associated with prejudice (racism: *r* = −0.08, *p* < 0.05; sexism: *r* = −0.05, n.s; ageism: *r* = −0.05, n.s; see Table 2). Although these findings were derived from a limited number of studies, they do offer further support for the notion that prejudice and tolerance are related but separate constructs (e.g., Butrus and Witenberg, 2013; van Doorn, 2014). Using different openness measures and examining how the facets of openness relate separately to prejudice and tolerance may offer future researchers an avenue to differentiate prejudice and tolerance empirically and conceptually.

As this review has illustrated, the few studies investigating the associations between the facets of openness and prejudice or tolerance do not show entirely consistent findings (i.e., there is heterogeneity in the effect size estimates). Nevertheless, the outcomes of our random effects meta-analysis indicate statistically significant associations across all models; that is, none of the 95% confidence intervals of the average effect size estimates across all six facets had contained zero. The discrepancies in the results from included studies were likely related to the variation in the types of outcome measures used (e.g., the use of the original Miville Guzman Universality-Diversity scale or the short form version), the variation in the kinds of prejudice and tolerance examined (e.g., racism, sexism, and ethnic prejudice), or variation in the definition of the prejudice and tolerance examined (e.g., sexism as hostile, benevolent, or subtle). Other differences include the different cultures in which the research was conducted, the different age groups represented in the samples, and the different openness measures used. From examining the literature, it is clear that more research using consistent, broad measures of prejudice (e.g., a generalized prejudice measure), and tolerance (e.g., Miville Guzman Universality-Diversity scale) are needed to elucidate the relationship between the facets of openness with prejudice and tolerance. In addition, future studies looking to summarize this area of research may consider conducting subgroup analysis on potential moderators (such as the types of outcome measures and the types of openness measure) and examine how these moderators may affect the associations between facets of openness and prejudice and tolerance.

### Lack of Non-Western Cultural Representation in the Current Literature

Almost all included studies were conducted using samples from Western cultures. Culture is a strong contextual factor that influenced the inner experience (e.g., how individual experiences and interprets social environment) as well as outward behaviors of an individual (e.g., how an individual behaves and interacts with others; Matsumoto et al., 2008; McDonald et al., 2011; Kende et al., 2018). Several studies had found contextual factors, such as culture, influenced both the development of personality (Allik and McCrae, 2004; Schmitt et al., 2007, 2008) and characteristic adaptations, such as intergroup behaviors and intergroup attitudes (Gerber et al., 2010; Kandler et al., 2012; Grijalva and Newman, 2015; Lee et al., 2018). Culture was also found to moderate the association of openness factor with intergroup bias and pro-diversity attitudes (e.g., Gerber et al., 2010; Alper and Yilmaz, 2019). For instance, the link between the openness factor and pro-diversity ideology is stronger among the participants from Western, educated, industrialized, rich, and democratic (WEIRD) cultures (Alper and Yilmaz, 2019). It is, therefore, very likely that contextual factors like culture moderate the relationship between the facets of openness and prejudice and tolerance. The facets of openness may influence prejudice and tolerance more strongly among participants from the WEIRD culture and weaker among participants from non-WEIRD culture. The relational pattern between the facets of openness and prejudice may also differ across cultures. The lack of cultural representation of non-Western settings represents a gap in the current literature and the generalizability of our findings. We also acknowledged that our search strategy of including only English articles likely inflated the numbers of WEIRD-biased samples. More research is needed to unravel the moderating effect of culture and examine the impact on the relationship between facets of openness and prejudice.

### Limitations of the Present Research

Our key contribution is in providing a descriptive numerical summary of the diverse literature on the association of facets of openness with prejudice and tolerance. As the focus of a scoping review lies in contextualizing current knowledge (i.e., identifying the current state of understanding, methodologies used, and gaps in understanding; Levac et al., 2010), this scoping review with meta-analyses highlighted the diverse findings by categorizing studies and their conclusion into themes. Although measures were taken to ensure that all relevant articles were captured by our search strategy (i.e., recruiting a library liaison officer to develop a search string and searching for gray literature using Google scholar and preprint database), and three major databases were used in our search strategy, only articles that were written in English were selected. Our search strategy and selection criteria may have caused us to lose relevant articles released in other databases or written in languages other than English. Using only publications written in English also likely inflates the numbers of WEIRD-based studies from our search strategy.

Despite using a broad search strategy, only 16 articles were identified as relevant from the existing literature. It is emphasized that the limited number of articles identified in this review represents a weakness of the current literature in providing a proper systematization of the evidence on the predictor-criterion relationship between the facets of openness and different forms of prejudice and tolerance, and not necessarily represents a weakness of our search strategy. Nevertheless, the limited number of studies summarized in this review restricted the generalizability of our conclusion. That is, the relations between the facets of openness and the constructs of prejudice and tolerance outlined in this review should be treated as preliminary. More studies are needed to validate (or invalidate) the associations of facets of openness with indices of prejudice and tolerance summarized in this paper before any firm conclusions can be made.

### Future Directions

Many psychologists have argued that using factor scores might obscure facet-criterion relationships (e.g., Hastings and O'Neill, 2009; Woo et al., 2014b). This review provides a more nuanced understanding of the facet-level associations between the openness factor and the different forms of prejudice and tolerance. As discussed, not all the facets of openness are associated equally with prejudice and tolerance. The associations reported in our review were based only on a few studies and thus should be treated as preliminary; future replication studies are needed to confirm the trends found in this review. As more evidence becomes available, an update in this review is encouraged to provide a timely guide for researchers interested in the link between openness facets and forms of prejudice and tolerance.

Recently, Woo et al. (2014b) had developed a new measure, the Six-Facet Openness Scale (SFOS), which covered most of the facets of openness unexamined by the NEO-PI-R. This new measure may complement NEO-PI-R in examining the full spectrum of openness facets. Other measures of the facets of openness beyond those discussed in this review may also be considered in future investigations to explore the broad range of openness facets and their relationship with prejudice and tolerance. For instance, the nine intellect scales in the Abridged Big Five Circumplex (AB5C; Hofstee et al., 1992), the homogenous item clusters of intellectance and school success in the Hogan Personality Inventory (Hogan et al., 1996), and the analytical item cluster in the Jackson Personality Inventory (Jackson, 1994) contain openness to intellectual pursuits not otherwise covered by the NEO-PI-R. For more definitive conclusions about the openness facets from different inventories and prejudice/tolerance, a large-scale study or series of studies in which all inventories assessing openness facets are included, and multiple kinds of prejudice and tolerance are assessed in different ways (including behavioral and implicit measures of prejudice), may be necessary.

More studies are also needed to examine the predictive utility of the facets of openness in prejudice and tolerance among non-Western cultures. The majority of the studies that examined the relationship between the facets of openness and prejudice or tolerance were conducted in western countries; no studies were identified from Africa, South America, and major parts of Asia (i.e., central, south, east, or south-east Asia). As discussed earlier, cultures might likely moderate the relationship between the facets of openness and prejudice. Alper and Yilmaz (2019) noted that the openness factor was strongly associated with Egalitarianism only when the presiding culture is liberal and does not oppress freedom of expression. Conversely, the openness factor was weakly linked with Egalitarianism when the culture was conservative and authoritarian. It is recommended for more studies to be conducted in non-Western countries (i.e., Africa, South America, and Asia) and for future studies to examine how different cultures (i.e., conservative cultures) affect the relationship between the facets of openness and prejudice or tolerance.

## Conclusion

This scoping review with meta-analysis provides a preliminary guide on the link between the facets of openness and different indices of prejudice or tolerance. This review is also the first study that systematically reviewed the relationship between the facets of openness and tolerance. Several gaps in the current literature were identified; future studies looking into the relationship between the facets of openness and prejudice or tolerance may consider following our recommendation and building on the gaps identified. Ultimately, this review adds to the growing research in prejudice and tolerance and contributes knowledge toward identifying the personality of prejudice and tolerance.

## Author Contributions

DN created research questions, conducted the article search, prepared the raw data, screened and analyzed the data, and prepared the manuscript. PL and NM screened the research articles and contributed to manuscript editing. KC provided advice on the statistical methods (i.e., meta-analysis) and contributed to manuscript editing. JR created research questions, screened the research articles, and contributed to manuscript editing. All authors contributed to the article and approved the submitted version.

## Funding

This research publication was funded by the Internal Research Fund, James Cook University, Singapore.

## Conflict of Interest

The authors declare that the research was conducted in the absence of any commercial or financial relationships that could be construed as a potential conflict of interest.

## Publisher's Note

All claims expressed in this article are solely those of the authors and do not necessarily represent those of their affiliated organizations, or those of the publisher, the editors and the reviewers. Any product that may be evaluated in this article, or claim that may be made by its manufacturer, is not guaranteed or endorsed by the publisher.

## References

[B1] Abrams D. (2010). Processes of Prejudice: Theory, Evidence, and Intervention (Research Report 56). Manchester: Equality and Human Rights Commission.

[B2] Ackermann K. Ackermann M. (2015). The Big Five in context: personality, diversity and attitudes toward equal opportunities for immigrants in Switzerland. Swiss Polit. Sci. Rev. 21, 396–418. 10.1111/spsr.12170

[B3] Agnew C. R. Thompson V. D. Smith V. A. Gramzow R. H. Currey D. P. (1993). Proximal and distal predictors of homophobia: framing the multivariate roots of outgroup rejection. J. Appl. Soc. Psychol. 23, 2013–2042. 10.1111/j.1559-1816.1993.tb01077.x

[B4] Akrami N. Ekehammar B. Araya T. (2000). Classical and modern racial prejudice: a study of attitudes toward immigrants in Sweden. Eur. J. Soc. Psychol. 30, 521–532. 10.1002/1099-0992(200007/08)30:4<521::AID-EJSP5>3.0.CO;2-N

[B5] Akrami N. Ekehammar B. Claesson M. Sonnander K. (2006). Classical and modern prejudice: attitudes toward people with intellectual disabilities. Res. Dev. Disabil. 27, 605–617. 10.1016/j.ridd.2005.07.003 16309887

[B6] Albrecht A. Dilchert S. Deller J. Paulus F. M. (2014). Openness in cross-cultural work settings: a multicountry study of expatriates. J. Pers. Assess. 96, 64–75. 10.1080/00223891.2013.821074 24003885

[B7] Allik J. McCrae R. R. (2004). Toward a geography of personality traits: patterns of profiles across 36 cultures. J. Cross Cult. Psychol. 35, 13–28. 10.1177/0022022103260382

[B8] Allport G. W. (1954). The Nature of Prejudice. Cambridge, MA: Perseus Books.

[B9] Alper S. Yilmaz O. (2019). How is the Big Five related to moral and political convictions: the moderating role of the WEIRDness of the culture. Pers. Individ. Dif. 145, 32–38. 10.1016/j.paid.2019.03.018

[B10] Amodio D. M. (2014). The neuroscience of prejudice and stereotyping. Nat. Rev. 15, 670–682. 10.1038/nrn3800 25186236

[B11] ^*^ Anglim J. Knowles E. R. V. Dunlop P. D. Marty A. (2017). HEXACO personality and Schwartz's personal values: a facet-level analysis. J. Res. Pers. 68, 23–31. 10.1016/j.jrp.2017.04.002

[B12] ^*^ Anglim J. Sojo V. Ashford L. J. Newman A. Marty A. (2019). Predicting employee attitudes to workplace diversity from personality, values, and cognitive ability. J. Res. Pers. 83:103865. 10.1016/j.jrp.2019.103865

[B13] Arksey H. O'Malley L. (2005). Scoping studies: towards a methodological framework. Int. J. Soc. Res. Methodol. 8, 19–32. 10.1080/1364557032000119616

[B14] Ashburn-Nardo L. Monteith M. J. Arthur S. A. Bain A. (2007). Race and the psychological health of African Americans. Group Proc. Intergroup Relat. 10, 471–491. 10.1177/1368430207081536

[B15] Ashton M. C. (1998). Personality and job performance: the importance of narrow traits. J. Organiz. Behav. 19, 289–303. 10.1002/(SICI)1099-1379(199805)19:3<289::AID-JOB841>3.0.CO;2-C

[B16] ^*^ Averhart V. (2012). Ageism in the Workplace: Examining the Influence of Age Conceptualization on the Advancement Opportunities of Older Workers. (Unpublished doctoral dissertation), Florida International University, Miami, FL, United States. 10.25148/etd.FI12050121

[B17] Bambulyaka M. (2011). The implicit methods for the study of tolerance. Int. J. Psychol. Behav. Sci. 5, 1888–1892. 10.5281/zenodo.1055972

[B18] Bartholow B. D. Dickter C. L. Sestir M. A. (2006). Stereotype activation and control of race bias: cognitive control of inhibition and its impairment by alcohol. J. Pers. Soc. Psychol. 90, 272–287. 10.1037/0022-3514.90.2.272 16536651

[B19] Batool M. Akram B. (2020). Development and validation of religious tolerance scale for youth. J. Relig. Health 59, 1481–1493. 10.1007/s10943-019-00897-5 31414338

[B20] Beer J. S. Stallen M. Lombardo M. V. Gonsalkorale K. Cunningham W. A. Sherman J. W. (2008). The quadruple process model approach to examining the neural underpinnings of prejudice. Neuroimage 43, 775–783. 10.1016/j.neuroimage.2008.08.033 18809502

[B21] Boies K. Lee K. Ashton M. C. Pascal S. Nicol A. A. M. (2001). The structure of the French personality lexicon. Eur. J. Pers. 15, 277–295. 10.1002/per.411

[B22] Brandt M. J. Chambers J. R. Crawford J. T. Wetherell G. Reyna C. (2015). Bounded openness: the effect of openness to experience on intolerance is moderated by target group conventionality. J. Pers. Soc. Psychol. 109, 549–568. 10.1037/pspp0000055 26167801

[B23] Butrus N. Witenberg R. T. (2013). Some personality predictors of tolerance to human diversity: the roles of openness, agreeableness, and empathy. Aust. Psychol. 48, 290–298. 10.1111/j.1742-9544.2012.00081.x

[B24] Carlucci L. Tommasi M. Saggino A. (2011). Socio-demographic and five factor model variables as predictors of religious fundamentalism: an italian study. Arch. Psychol. Religion 33, 253–268. 10.1163/157361211X576609

[B25] Cattell H. E. P. Mead A. D. (2008). The sixteen personality factor questionnaire (16PF), in The SAGE Handbook of Personality Theory and Assessment, Vol. 2, eds Boyle G. J. Matthews G. Saklofske D. H. (London: SAGE Publications), 135–159.

[B26] Chekroud A. M. Everett J. A. C. Bridge H. Hewstone M. (2014). A review of neuroimaging studies of race-related prejudice: Does amygdala response reflect threat? Front. Hum. Neurosci. 8:179. 10.3389/fnhum.2014.00179 24734016 PMC3973920

[B27] Christensen A. P. Cotter K. N. Silvia P. J. (2019). Reopening openness to experience: a network analysis of four openness to experience inventories. J. Pers. Assess. 101, 574–588. 10.1080/00223891.2018.1467428 29746176

[B28] ^*^ Christopher A. N. Zabel K. L. Miller D. E. (2013). Personality, authoritarianism, social dominance, and ambivalent sexism: a mediational model. Individ. Diff. Res. 11, 70-80. Available online at: https://www.researchgate.net/publication/289189142_Personality_authoritarianism_social_dominance_and_ambivalent_sexism_A_mediational_model (accessed October 31, 2020).

[B29] Clark J. Glasziou P. Del Mar C. Bannach-Brown A. Stehlik P. Scott A. M. (2020). A full systematic review was completed in 2 weeks using automation tools: a case study. J. Clin. Epidemiol. 121, 81–90. 10.1016/j.jclinepi.2020.01.008 32004673

[B30] Connelly B. S. Ones D. S. Davies S. E. Birkland A. (2014). Opening up openness: a theoretical sort following critical incidents methodology and a meta-analytic investigation of the trait family measures. J. Pers. Assess. 96, 17–28. 10.1080/00223891.2013.809355 23819531

[B31] Costa P. T. Jr. McCrae R. R. (1992). Revised NEO Personality Inventory (NEO-PI-R) and NEO Five Factor Inventory (NEO-FFI) professional manual. Odessa, FL: Psychological Assessment Resources.

[B32] Costa P. T. Jr. McCrae R. R. (2008). The revised NEO personality inventory, in The SAGE Handbook of Personality Theory and Assessment, Vol. 2, eds Boyle G. J. Matthews G. Saklofske D. H. (London: SAGE Publications), 179–198.

[B33] Cote R. R. Erickson B. H. (2009). Untangling the roots of tolerance: How forms of social capital shape attitudes toward ethnic minorities and immigrants. Am. Behav. Sci. 52, 1664–1689. 10.1177/0002764209331532

[B34] Crawford J. T. (2014). Ideological symmetries and asymmetries in political intolerance and prejudice toward political activist groups. J. Exp. Soc. Psychol. 55, 284–298. 10.1016/j.jesp.2014.08.002

[B35] Crawford J. T. Brandt M. J. (2019). Who is prejudiced, and toward whom? The Big Five traits and generalized prejudice. Pers. Soc. Psychol. Bull. 45, 1455–1467. 10.1177/0146167219832335 30895844

[B36] Cvencek D. Greenwald A. Brown A. Gray N. Snowden R. (2010). Faking of the Implicit Association Test is statistically detectable and partly correctable. Basic Appl. Soc. Psych. 32, 302–214. 10.1080/01973533.2010.519236

[B37] de Raad B. van Heck G. L. (1994). Editorial special issue: the fifth of the Big Five. Eur. J. Pers. 8, 225–227. 10.1002/per.2410080402

[B38] Devine P. G. Plant E. A. Amodio D. M. Harmon-Jones E. Vance S. L. (2002). The regulation of explicit and implicit race bias: the role of motivations to respond without prejudice. J. Pers. Soc. Psychol. 82, 835–848. 10.1037/0022-3514.82.5.835 12003481

[B39] DeYoung C. G. (2006). Higher-order factors of the Big Five in a multi-informant sample. J. Pers. Soc. Psychol. 91, 1138–1151. 10.1037/0022-3514.91.6.1138 17144770

[B40] DeYoung C. G. Quilty L. C. Peterson J. B. (2007). Between facets and domains: 10 aspects of the Big Five. J. Pers. Soc. Psychol. 93, 880–896. 10.1037/0022-3514.93.5.880 17983306

[B41] Digman J. M. (1990). Personality structure: emergence of the Five-Factor Model. Annu. Rev. Psychol. 41, 417–440. 10.1146/annurev.ps.41.020190.002221

[B42] Digman J. M. (1997). Higher-order factors of the Big Five. J. Pers. Soc. Psychol. 73, 1246–1256. 10.1037/0022-3514.73.6.1246 9418278

[B43] Dover T. L. Hunger J. M. Major B. (2020). Health consequences of prejudice and discrimination, in The Wiley Encyclopedia of Health Psychology, eds Sweeny K. Robbins M. L. Cohen L. M. (Hoboken, NJ: John Wiley and Sons Ltd.), 231–238. 10.1002/9781119057840.ch71

[B44] Duckitt J. (1992). Psychology and Prejudice: a historical analysis and integrative framework. Am. Psychol. 47, 1182–1193. 10.1037/0003-066X.47.10.1182

[B45] Duckitt J. (2019). The Social Psychology of Prejudice. New York, NY: Praeger Publishers.

[B46] Duriez B. Soenens B. (2006). Personality, identity styles and authoritarianism: an integrative study among late adolescents. Eur. J. Pers. 20, 397–417. 10.1002/per.589 15335331

[B47] Duriez B. Van Hiel A. (2002). The march of modern fascism. A comparison of social dominance orientation and authoritarianism. Personal. Individ. Diff. 32, 1199–1213. 10.1016/S0191-886900086-1

[B48] Egger M. Davey Smith G. Schneider M. Minder C. (1997). Bias in meta-analysis detected by a simple, graphical test. BMJ 315, 629–634. 10.1136/bmj.315.7109.629 9310563 PMC2127453

[B49] ^*^ Ekehammar B. Akrami N. (2007). Personality and prejudice: from Big Five personality factors to facets. J. Pers. 75, 899–925. 10.1111/j.1467-6494.2007.00460.x 17760851

[B50] Ekehammar B. Akrami N. Araya T. (2000). Development and validation of Swedish classical and modern sexism scales. Scand. J. Psychol. 41, 307–314. 10.1111/1467-9450.00203 11131952

[B51] Elleman L. G. Condon D. M. Holtzman N. S. Allen V. R. Revelle W. (2020). Smaller is better: associations between personality and demographics are improved by examining narrower traits and regions. Collabra Psychol. 6:17210. 10.1525/collabra.17210

[B52] Fisher Z. Tipton E. (2015). Robumeta: Robust Variance Meta-regression. R Package Version 1.6. Available online at: http://CRAN.R-project.org/package=robumeta (accessed June 31, 2021).

[B53] Flynn F. J. (2005). Having an open mind: the impact of openness to experience on interracial attitudes and impression formation. J. Pers. Soc. Psychol. 88, 816–826. 10.1037/0022-3514.88.5.816 15898877

[B54] Fraboni M. Saltstone R. Hughes S. (1990). The Fraboni Scale of Ageism (FSA): an attempt at more precise measure of ageism. Can. J. Aging 9, 56–66. 10.1017/S0714980800016093

[B55] Freitag M. Rapp C. (2015). The personal foundations of political tolerance towards immigrants. J. Ethn. Migr. Stud. 41, 351–373. 10.1080/1369183X.2014.924847

[B56] Fuertes J. N. Miville M. L. Mohr J. J. Sedlacek W. E. Gretchen D. (2000). Factor structure and short form of the miville-guzman universality-diversity scale. Measur. Eval. Counsel. Dev. 33, 157–169. 10.1080/07481756.2000.1206900725045958

[B57] Geoffrey B. (2013). Our Racist Heart: An Exploration of Unconscious Prejudice in Everyday Life. London: Routledge.

[B58] Gerber A. S. Huber G. A. Doherty D. Dowling C. M. Ha S. E. (2010). Personality and political attitudes: relationship across issue domains and political contexts. Am. Polit. Sci. Rev. 104, 111–133. 10.1017/S0003055410000031

[B59] Gibson J. L. (1992). Alternative measures of political tolerance – must tolerance be ‘least-liked’? Am. J. Polit. Sci. 36, 560–577. 10.2307/2111491

[B60] Glick P. Fiske S. T. (1996). The ambivalent sexism inventory: differentiating hostile and benevolent sexism. J. Personal. Soc. Psychol. 70, 491–512. 10.1037/0022-3514.70.3.491

[B61] Goldberg L. R. (1990). An alternative “description of personality”: the Big-Five factor structure. J. Pers. Soc. Psychol. 59, 1216–1229. 10.1037/0022-3514.59.6.1216 2283588

[B62] Goldberg L. R. (1993). The structure of phenotypic personality traits. Am. Psychol. 48:26. 10.1037/0003-066X.48.1.26 8427480

[B63] Goldberg L. R. (1999). A broad-bandwidth, public domain, personality inventory measuring the lower-level facets of several five-factor models, in Personality Psychology in Europe, Vol. 7, eds Mervielde I. Deary I. De Fruyt F. Ostendorf F. (Tilburg: Tilburg University Press), 7–28.

[B64] Greenwald A. McGhee D. Schwartz J. (1998). Measuring individual differences in implicit cognition: the implicit association test. J. Pers. Soc. Psychol. 74, 1464–1480. 10.1037/0022-3514.74.6.1464 9654756

[B65] Griffin B. Hesketh B. (2004). Why openness to experience is not a good predictor of job performance. Int. J. Select. Assess. 12, 243–251. 10.1111/j.0965-075X.2004.278_1.x

[B66] Grijalva E. Newman D. A. (2015). Narcissism and counterproductive work behavior: meta-analysis and consideration of collectivist culture, Big Five personality, and Narcissism's facet structure. Appl. Psychol. 64, 93–126. 10.1111/apps.12025

[B67] Hamer K. McFarland S. Penczek M. (2019). What lies beneath? Predictors of identification with all humanity. Pers. Individ. Differ. 141, 258–267. 10.1016/j.paid.2018.12.019

[B68] ^*^ Han S. Pistole M. C. (2017). Big Five personality factors and facets as predictors of openness to diversity. J. Psychol. 151, 752–766. 10.1080/00223980.2017.1393377 29166225

[B69] Harris R. Cormack D. Tobias M. Yeh L. Talamaivao N. Minster J.. (2012). The pervasive effects of racism: experiences of racial discrimination in New Zealand over time and associations with multiple health domains. Soc. Sci. Med. 74, 408–415. 10.1016/j.socscimed.2011.11.004 22204840

[B70] Hastings S. E. O'Neill T. A. (2009). Predicting workplace deviance using broad versus narrow personality variables. Pers. Individ. Dif. 47, 289–293. 10.1016/j.paid.2009.03.015

[B71] Herek G. M. (1988). Heterosexuals' attitudes toward lesbians and gay men: correlates and gender differences. J. Sex Res. 25, 451–477. 10.1080/00224498809551476

[B72] Higgins J. P. T. Thompson S. G. Deeks J. J. Altman D. G. (2003). Measuring inconsistency in meta-analyses. BMJ 327, 557–560. 10.1136/bmj.327.7414.557 12958120 PMC192859

[B73] Hjerm M. (2005). What the future may bring. Acta Sociol. 48, 292–307. 10.1177/0001699305059943

[B74] Hjerm M. Eger M. A. Bohman A. Connolly F. F. (2019). A new approach to the study of tolerance: conceptualizing and measuring acceptance, respect, and appreciation of difference. Soc. Indic. Res. 147, 897–919. 10.1007/s11205-019-02176-y

[B75] Hodson G. Dhont K. (2015). The person-based nature of prejudice: Individual difference predictors of intergroup negativity. Eur. Rev. Soc. Psychol. 26, 1–42. 10.1080/10463283.2015.1070018

[B76] Hofstee W. K. de Raad B. Goldberg L. R. (1992). Integration of the Big Five and circumplex approaches to trait structure. J. Pers. Soc. Psychol. 63, 146–163. 10.1037/0022-3514.63.1.146 1494982

[B77] Hogan J. Brinkmeyer K. Hogan R. (1996). Hogan Personality Inventory Form Manual. Tulsa, OK: Hogan Assessment Systems.

[B78] Hough L. M. Ones D. S. (2001). The structure, measurement, validity, and use of personality variables in industrial, work, and organizational psychology, in Handbook of Industrial, Work and Organizational Psychology - Volume 1: Personnel Psychology, Vol. 2, eds Anderson N. Ones D. S. Sinangil H. K. Viswesvaran C. (London: SAGE Publications Ltd.), 233–277. 10.4135/9781848608320.n13

[B79] ^*^ Huxley E. Bizumic B. Kenny A. (2015). The Role of Ethnocentrism in the Relationship Between Openness to Experience and Ethnic Prejudice. Ethnic and Cultural Identity: Perceptions, Discrimination and Social Challenges. New York, NY: Nova Science, 85–101. Available online at: https://www.academia.edu/44639379/The_role_of_ethnocentrism_in_the_relationship_between_openness_to_experience_and_ethnic_prejudice?from=cover_page (accessed May 12, 2021).

[B80] Jackson D. N. (1994). Jackson Personality Inventory-Revised Manual. Port Huron, MI: Research Psychologists Press.

[B81] John O. P. Srivastava S. (1999). The Big Five trait taxonomy: History, measurement, and theoretical perspectives, in Handbook of Personality: Theory and Research, eds Pervin L. A. John O. P. (New York, NY: Guilford Press), 102–138.

[B82] Judge T. A. Rodell J. B. Klinger R. L. Simon L. S. Crawford E. R. (2013). Hierarchical representations of the five-factor model of personality in predicting job performance: integrating three organizing frameworks with two theoretical perspectives. J. Appl. Psychol. 98, 875–925. 10.1037/a0033901 24016206

[B83] ^*^ Kandler C. Bleidorn W. Riemann R. (2012). Left or right? Sources of political orientation: the roles of genetic factors, cultural transmission, assortative mating, and personality. J. Pers. Soc. Psychol. 102, 633–645. 10.1037/a0025560 21988277

[B84] Kende J. Phalet K. Van den Noortgate W. Kara A. Fischer R. (2018). Equality revisited: a cultural meta-analysis of intergroup contact and prejudice. Soc. Psychol. Pers. Sci. 9, 887–895. 10.1177/1948550617728993

[B85] Konstantopoulos S. (2011). Fixed effects and variance components estimation in three-level metaanalysis. Res. Synth. Methods 2, 61–76. 10.1002/jrsm.35 26061600

[B86] Lee K. Ashton M. C. (2004). Psychometric properties of the HEXACO personality inventory. Multivariate Behav. Res. 39, 329–358. 10.1207/s15327906mbr3902_8 26804579

[B87] Lee K. Ashton M. C. Griep Y. Edmonds M. (2018). Personality, religion, and politics: an investigation in 33 countries. Eur. J. Pers. 32, 100–115. 10.1002/per.2142

[B88] Legault L. Green-Demers I. Grant P. Chung J. (2007). On the self-regulation of implicit and explicit prejudice: a self-determination theory perspective. Pers. Soc. Psychol. Bull. 33, 732–749. 10.1177/0146167206298564 17440201

[B89] Leone L. Desimoni M. Chirumbolo A. (2012). HEXACO, social worldviews and socio-political attitudes: a mediation analysis. Pers. Individ. Dif. 53, 995–1001. 10.1016/j.paid.2012.07.016

[B90] Levac D. Colquhoun H. O'Brien K. (2010). Scoping studies: advancing the methodology. Implement. Sci. 5:69. 10.1186/1748-5908-5-69 20854677 PMC2954944

[B91] Liao H. Hong Y. Rounds J. (2016). Perception of subtle racism: the role of group status and legitimizing ideologies. Couns. Psychol. 44, 237–266. 10.1177/0011000015625329

[B92] Maples J. L. Guan L. Carter N. T. Miller J. D. (2014). A test of the international personality item pool representation of the revised NEO personality inventory and development of a 120-item IPIP-based measure of the five-factor model. Psychol. Assess. 26, 1070–1084. 10.1037/pas0000004 24932643

[B93] Matsumoto D. Yoo S. H. Fontaine J. (2008). Mapping expressive differences around the world: the relationship between emotional display rules and individualism versus collectivism. J. Cross Cult. Psychol. 39, 55–74. 10.1177/0022022107311854

[B94] McCord D. M. (2002). M5 Questionnaire (Available online at: mccord@email.wcu.edu).

[B95] McCrae R. R. Costa J. P. T. Martin T. A. (2005). The NEO–PI−3: a more readable revised NEO personality inventory. J. Pers. Assess. 84, 261–270. 10.1207/s15327752jpa8403_05 15907162

[B96] McCrae R. R. Costa P. T. (1987). Validation of the five-factor model of personality across instruments and observers. J. Pers. Soc. Psychol. 52, 81–90. 10.1037/0022-3514.52.1.81 3820081

[B97] McDonald M. M. Navarrete C. D. Sidanius J. (2011). Developing a theory of gendered prejudice: an evolutionary and social dominance perspective, in Social Cognition, Social Identity, and Intergroup Relations. eds Kramer R. M. Leonardelli G. L. Livingston R. W. (London: Taylor and Francis Group), 189–220.

[B98] Miklikowska M. (2015). Like parent, like child? Development of prejudice and tolerance towards immigrants. Br. J. Psychol. 107, 95–116. 10.1111/bjop.12124 25702782

[B99] ^*^ Miller A. K. (2019). A dilemma of dogma: specifying the personality root of sexual prejudice. J. Homosex. 68, 3–22. 10.1080/00918369.2019.1624454 31251701

[B100] ^*^ Miller A. K. Wagner M. M. Hunt A. N. (2012). Parsimony in personality: predicting sexual prejudice. J. Homosex. 59, 201–214. 10.1080/00918369.2012.638550 22335418

[B101] Miville M. L. Gelso C. J. Pannu R. Liu W. Touradji P. Fuertes J. (1999). Appreciating similarities and valuing differences: the Miville-Guzman Universality-Diversity scale. J. Couns. Psychol. 46, 291–307. 10.1037/0022-0167.46.3.291

[B102] Montei M. S. Adams G. A. Eggers L. M. (1996). Validity of scores on the attitudes toward diversity scale (ATDS). Educ. Psychol. Measur. 56, 293–303. 10.1177/0013164496056002010

[B103] Morley K. M. (2003). Fitting in by race/ethnicity: the social and academic integration of diverse students at a large predominantly white university. J. Coll. Stud. Retenti. Res. Theor. Pract. 5, 147–174. 10.2190/K1KF-RTLW-1DPW-T4CC

[B104] Mount M. K. Barrick M. R. Scullen S. M. Rounds J. (2005). Higher-order dimensions of the big five personality traits and the big six vocational interest types. Pers. Psychol. 58, 447–478. 10.1111/j.1744-6570.2005.00468.x

[B105] Mussel P. Winter C. Gelleri P. Schuler H. (2011). Explicating the openness to experience construct and its subdimensions and facets in a work setting. Int. J. Select. Assess. 19, 145–156. 10.1111/j.1468-2389.2011.00542.x

[B106] Noh S. Kaspar V. Wickrama K. A. S. (2007). Overt and subtle racial discrimination and mental health: preliminary findings of Korean immigrants. Am. J. Public Health 97, 1269–1274. 10.2105/AJPH.2005.085316 17538066 PMC1913092

[B107] Norman R. M. G. Sorrentino R. M. Windell D. Manchanda R. (2008). Are personal values of importance in the stigmatization of people with mental illness? Can. J. Psychiatr. 53, 848–886. 10.1177/070674370805301210 19087483

[B108] Norman W. T. (1963). Toward an adequate taxonomy of personality attributes: replicated factor structure in peer nomination personality ratings. J. Abnorm. Soc. Psychol. 66, 574–583. 10.1037/h0040291 13938947

[B109] Nosek B. Smyth F. Hansen J. Devos T. Lindner N. Ranganath K.. (2007). Pervasiveness and correlates of implicit attitudes and stereotypes. Eur. Rev. Soc. Psychol. 18, 36–88. 10.1080/10463280701489053

[B110] ^*^ Onraet E. Van Hiel A. Roets A. Cornelis I. (2011). The closed mind: ‘experience' and ‘cognition’ aspects of openness to experience and need for closure as psychological bases for right–wing attitudes. Eur. J. Pers. 25, 184–197. 10.1002/per.775

[B111] Oskarsson S. Widmalm S. (2016). Personality and political tolerance: evidence from India and Pakistan. Polit. Stud. 64, 235–254. 10.1111/1467-9248.12169

[B112] Ouzzani M. Hammady H. Fedorowicz Z. Elmagarmid A. (2016). Rayyan—a web and mobile app for systematic reviews. Syst. Rev. 5:210. 10.1186/s13643-016-0384-4 27919275 PMC5139140

[B113] Ozer D. J. (1985). Correlation and the coefficient of determination. Psychol. Bull. 97, 307–315. 10.1037/0033-2909.97.2.307

[B114] Paradies Y. Ben J. Denson N. Elias A. Priest N. Pieterse A.. (2015). Racism as a determinant of health: a systematic review and meta-analysis. PLoS ONE 10:e0138511. 10.1371/journal.pone.0138511 26398658 PMC4580597

[B115] Paunonen S. V. Rothstein M. G. Jackson D. N. (1999). Narrow reasoning about the use of broad personality measures for personnel selection. J. Organiz. Behav. 20, 389–405. 10.1002/(SICI)1099-1379(199905)20:3<389::AID-JOB917>3.0.CO;2-G

[B116] Peters M. D. J. Godfrey C. McInerney P. Munn Z. Tricco A. C. Khalil H. (2020). Chapter 11: scoping reviews, in JBI Manual for Evidence Synthesis, eds E. Aromataris, and Z. Munn. Available online at: https://synthesismanual.jbi.global (accessed September 30, 2020).

[B117] Peterson J. Pearce P. F. Ferguson L. A. Langford C. A. (2017). Understanding scoping reviews: definition, purpose, and process. J. Am. Assoc. Nurse Pract. 29, 12–16. 10.1002/2327-6924.12380 27245885

[B118] Pettigrew T. F. Meertens R. W. (1995). Subtle and blatant prejudice in western Europe. Eur. J. Soc. Psychol. 25, 57–75. 10.1002/ejsp.2420250106

[B119] Pham M. T. Rajic A. Greig J. D. Sargeant J. M. Papadopoulos A. McEwen S. A. (2014). A scoping review of scoping reviews: advancing the approach and enhancing the consistency. Res. Synth. Methods 5, 371–385. 10.1002/jrsm.1123 26052958 PMC4491356

[B120] Plaut V. C. (2010). Diversity science: why and how difference makes a difference. Psychol. Inq. 21, 77–99. 10.1080/10478401003676501

[B121] Pletzer J. L. Oostrom J. K. Bentvelzen M. de Vries R. E. (2020). Comparing domain- and facet-level relations of the HEXACO personality model with workplace deviance: a meta-analysis. Pers. Individ. Differ. 152:109539. 10.1016/j.paid.2019.109539

[B122] Pohan C. A. Aguilar T. E. (2001). Measuring educators' beliefs about diversity in personal and professional contexts. Am. Educ. Res. J. 38, 159–182. Available online at: https://www.researchgate.net/publication/35784026 (accessed September 6, 2021).

[B123] Polanin J. R. Pigott T. D. Espelage D. L. Grotpeter J. K. (2019). Best practice guidelines for abstract screening large-evidence systematic reviews and meta-analyses. Res. Synth. Method. 10, 330–342. 10.1002/jrsm.1354

[B124] ^*^ Proctor S. L. McCord D. M. (2009). Correlates of the openness to experience domain. Individ. Diff. Res. 7, 222-227. Available online at: https://www.researchgate.net/publication/259503648_Correlates_of_the_Openness_to_Experience_Domain (accessed October 31, 2020).

[B125] Quintana D. S. (2015). From pre-registration to publication: a non-technical primer for conducting a meta-analysis to synthesize correlational data. Front. Psychol. 6:1549. 10.3389/fpsyg.2015.01549 26500598 PMC4597034

[B126] R Development Core Team (2015). R: A Language and Environment for Statistical Computing. Vienna: The R Foundation for Statistical Computing.

[B127] Rapp C. Freitag M. (2015). Teaching tolerance? Associational diversity and tolerance formation. Polit. Stud. 63, 1031–1051. 10.1111/1467-9248.12142

[B128] Riley R. D. Higgins J. P. T. Deeks J. J. (2011). Interpretation of random effects meta-analyses. BMJ 342:d549. 10.1136/bmj.d549 21310794

[B129] ^*^ Roccas S. Sagiv L. Schwartz S. H. Knafo A. (2002). The Big Five personality factors and personal values. Pers. Soc. Psychol. Bull. 28, 789–801. 10.1177/0146167202289008

[B130] Rushton J. P. Irwing P. (2008). A general factor of personality (GFP) from two meta-analyses of the Big Five: Digman (1997) and Mount, Barrick, Scullen, and Rounds (2005). Pers. Individ. Dif. 45, 679–683. 10.1016/j.paid.2008.07.015

[B131] Saef R. M. Porter C. M. Woo S. E. Wiese C. (2019). Getting off on the right foot: the role of openness to experience in fostering initial trust between culturally dissimilar partners. J. Res. Pers. 79, 176–187. 10.1016/j.jrp.2019.03.003

[B132] Saucier G. Ostendorf F. (1999). Hierarchical subcomponents of the Big Five personality factors: a cross-language replication. J. Pers. Soc. Psychol. 76, 613–627. 10.1037/0022-3514.76.4.613 10234848

[B133] Schmitt D. P. Allik J. McCrae R. R. Benet-Martinez V. (2007). The geographic distribution of Big Five personality traits: patterns and profiles of human self-description across 56 nations. J. Cross Cult. Psychol. 38, 173–212. 10.1177/0022022106297299

[B134] Schmitt D. P. Realo A. Voracek M. Allik J. (2008). Why can't a man be more like a woman? Sex differences in Big Five personality traits across 55 cultures. J. Pers. Soc. Psychol. 94, 168–182. 10.1037/0022-3514.94.1.168 18179326

[B135] Schwaba T. Rhemtulla M. Hopwood C. J. Bleidorn W. (2020). A facet atlas: visualizing networks that describe the blends, cores, and peripheries of personality structure. PLoS ONE 15:e0236893. 10.1371/journal.pone.0236893 32730328 PMC7392538

[B136] Schwartz S. H. (1992). Universals in the content and structure of values: theoretical advances and empirical tests in 20 countries, in Advances in Experimental Social Psychology. Vol. 25, ed Zanna M. P. (New York, NY. Academic Press), 1–65.

[B137] Schwartz S. H. Cieciuch J. Vecchione M. Davidov E. Fischer R. Beierlein C.. (2012). Refining the theory of basic individual values. J. Pers. Soc. Psychol. 103, 663–688. 10.1037/a0029393 22823292

[B138] Sibley C. G. Duckitt J. (2008). Personality and prejudice: a meta-analysis and theoretical review. Pers. Soc. Psychol. Rev. 12, 248–279. 10.1177/1088868308319226 18641385

[B139] Sibley C. G. Duckitt J. (2010). Personality geneses of authoritarianism: the form and function of openness to experience, in Perspectives on Authoritarianism, eds Funke F. Petzel Th. Cohrs J. C. Duckitt J. (Wiesbaden: VS-Verlag), 169–199. 10.13140/RG.2.1.2640.2724

[B140] Sibley C. G. Duckitt J. (2013). The dual process model of ideology and prejudice: a longitudinal test during a global recession. J. Soc. Psychol. 153, 448–466. 10.1080/00224545.2012.757544 23951951

[B141] Sibley C. G. Osborne D. Duckitt J. (2012). Personality and political orientation: meta-analysis and test of a threat-constraint model. J. Res. Pers. 46, 664–677. 10.1016/j.jrp.2012.08.002

[B142] Son Hing L. S. Chung-Yan G. A. Hamilton L. K. Zanna M. P. (2008). A two-dimensional model that employs explicit and implicit attitudes to characterized prejudice. J. Pers. Soc. Psychol. 94, 971–987. 10.1037/0022-3514.94.6.971 18505312

[B143] Son Hing L. S. Zanna M. P. (2010). Individual differences, in The SAGE Handbook of Prejudice, Stereotyping and Discrimination, eds Dovidio J. F. Hewstone M. Glick P. Esses V. M. (London: SAGE Publications Ltd.), 163–178. 10.4135/9781446200919.n10

[B144] Song F. Sheldon T. A. Sutton A. J. Abrams K. R. Jones D. R. (2001). Methods for exploring heterogeneity in meta-analysis. Eval. Health Profess. 24, 126–151. 10.1177/016327870102400203 11523383

[B145] Sparkman D. J. Eidelman S. Dueweke A. R. Marin M. S. Dominguez B. (2019). Open to diversity openness to experience predicts beliefs in multiculturalism and colorblindness through perspective taking. J. Individ. Differ. 40, 1–12. 10.1027/1614-0001/a000270

[B146] Sturmer S. Benbow A. E. F. Siem B. Barth M. Bodansky A. N. Lotz-Schmitt K. (2013). Psychological foundations of xenophilia: the role of major personality traits in predicting favorable attitudes toward cross-cultural contact and exploration. J. Pers. Soc. Psychol. 105, 832–851. 10.1037/a0033488 23834640

[B147] Subbaraman N. (2020). Grieving and frustrated: black scientists call out racism in the wake of police killings. Nature 582, 155–156. 10.1038/d41586-020-01705-x 32518341

[B148] ^*^ Szeto A. C. H. O'Neill T. A. Dobson K. S. (2015). The association between personality and individual differences and stigma toward people with mental disorders. Am. J. Psychiatr. Rehabil. 18, 303–332. 10.1080/15487768.2015.1089799

[B149] ^*^ Thompson R. L. Brossart D. F. Carlozzi A. F. Miville M. L. (2002). Five-factor model (Big Five) personality traits and universal-diverse orientation in counselor trainees. J. Psychol. 136, 561–572. 10.1080/00223980209605551 12431039

[B150] Tricco A. C. Lillie E. Zarin W. O'Brien K. K. Colquhoun H. Levac D.. (2018). PRISMA extension for scoping reviews (PRISMA-ScR): checklist and explanation. Ann. Intern. Med. 169, 467–473. 10.7326/M18-0850 30178033

[B151] Tropp L. R. (2003). The psychological impact of prejudice: implications for intergroup contact. Group Proc. Intergroup Relat. 6, 131–149. 10.1177/136843020300600200119302731

[B152] ^*^ Unruh L. E. McCord D. M. (2010). Personality traits and beliefs about diversity in pre-service teachers. Individ. Diff. Res. 8, 1–7. Available online at: https://www.researchgate.net/publication/259503486_Personality_Traits_and_Beliefs_About_Diversity

[B153] Van der Noll J. Poppe E. Verkuyten M. (2010). Political tolerance and prejudice: differential reactions toward muslims in the Netherlands. Basic Appl. Soc. Psych. 32, 46–56. 10.1080/01973530903540067

[B154] Van der Walt J. L. (2014). Measuring Religious Tolerance in Education. Retrieved from: https://www.driestar-educatief.nl/medialibrary/Driestar/Engelse-website/Documenten/2014-VanderWalt-Measuring-religious-tolerance-in-education.pdf (accessed November 16, 2020).

[B155] van Doorn M. (2014). The nature of tolerance and the social circumstances in which it emerges. Curr. Sociol. Rev. 62, 905–927. 10.1177/0011392114537281

[B156] Van Hiel A. Mervielde I. (1996). Personality and current political beliefs. Psychol. Belgica 36, 221–226. 10.5334/pb.902

[B157] van Zalk M. H. W. Kerr M. (2014). Developmental trajectories of prejudice and tolerance toward immigrants from early to late adolescence. J. Youth Adolesc. 43, 1658–1671. 10.1007/s10964-014-0164-1 25138528

[B158] Vasiljevic M. Crisp R. J. (2013). Tolerance by surprise: evidence for a generalized reduction in prejudice and increased egalitarianism through novel category combination. PLoS ONE 8:e57106. 10.1371/journal.pone.0057106 23483895 PMC3590200

[B159] Verkuyten M. Slooter L. (2007). Tolerance of Muslim beliefs and practices: age related differences and context effects. Int. J. Behav. Dev. 31, 467–477. 10.1177/0165025407081480

[B160] Viechtbauer W. (2010). Conducting meta-analyses in R with the metafor package. J. Stat. Softw. 36, 1–48. 10.18637/jss.v036.i03

[B161] Viechtbauer W. Cheung M. W. (2010). Outlier and influence diagnostics for meta-analysis. Res. Synth. Methods 1, 112–125. 10.1002/jrsm.11 26061377

[B162] Vogt W. P. (1994). What Is Tolerance and Why Should We Teach It? Rev. Educ. Pedag. Cult. Stud. 16, 277–296. 10.1080/1071441940160304

[B163] Weatherford R. D. Spokane A. R. (2013). The relationship between personality dispositions, multicultural exposure, and multicultural case conceptualization ability. Train. Educ. Prof. Psychol. 7, 215–224. 10.1037/a0033543

[B164] Williams D. R. (2018). Stress and the Mental Health of Populations of color: advancing our understanding of race-related stressors. J. Health Soc. Behav. 59, 466–485. 10.1177/0022146518814251 30484715 PMC6532404

[B165] Witenberg R. T. (2007). The moral dimension of children's and adolescents'conceptualization of tolerance to human diversity. J. Moral Educ. 36, 433–451. 10.1080/03057240701688002

[B166] Woo S. E. Chernyshenko O. S. Longley A. Zhang Z. X. Chiu C. Y. Stark S. E. (2014a). Openness to experience: its lower level structure, measurement, and cross-cultural equivalence. J. Pers. Assess. 96, 29–45. 10.1080/00223891.2013.806328 23795950

[B167] Woo S. E. Chernyshenko O. S. Stark S. E. Conz G. (2014b). Validity of six openness facets in predicting work behaviors: a meta-analysis. J. Pers. Assess. 96, 76–86. 10.1080/00223891.2013.806329 23795997

