# Supplementary material for: Associations Between Openness Facets, Prejudice, and Tolerance: A Scoping Review With Meta-Analysis
Source: Front Psychol. 2021 Sep 28;12:707652. doi: 10.3389/fpsyg.2021.707652 (PMC8506218; doi:10.3389/fpsyg.2021.707652)
Supplement: Supplementary file 1 [file Table_1.DOCX]

Supplementary Material

Associations between Openness Facets, Prejudice, and Tolerance: A Scoping Review

Da Xuan Ng^*^, Patrick K. F. Lin, Nigel V. Marsh, and Jonathan E. Ramsay

^1^School of Social and Health Sciences, James Cook University, Singapore

*** Correspondence:** Da Xuan Ng, da.ng@my.jcu.edu.au

# Supplementary Figures

## Supplementary Figure 1. Title and Abstract Screening Tool.

1. Is the citation written in English?

| - Yes, ( proceed) | - No, exclude ( screen next article) |
| --- | --- |

1. Is the citation type classified under any of these categories?

- Thesis
- Journal articles
- Conference papers
- Book or Book chapters

| - Yes, ( proceed) | - Unsure ( proceed) | - No, exclude ( screen next article) |
| --- | --- | --- |
|  |  |  |

1. Is the study primary research? (i.e., NOT opinion papers, theoretical papers or lecture notes)

| - Yes, ( proceed) | - Unsure ( proceed) | - No, exclude ( screen next article) |
| --- | --- | --- |
|  |  |  |

1. Does the abstract or title mentions the use of personality measure? (related terms include: NEO-PI, BFI, HEXACO, IPIP)

| - Yes, ( proceed) | - Unsure ( proceed) | - No, exclude ( screen next article) |
| --- | --- | --- |

1. Does the abstract or title mentions Openness to Experience and/or its facets? (related terms include: Intellect, Openness, Experiencing, “Big five”, “five factor”)

| - Yes, ( proceed) | - Unsure ( proceed) | - No, exclude ( screen next article) |
| --- | --- | --- |

1. Does the abstract or title mentions terms related to prejudice or diversity tolerance? (related terms include: intergroup relations, stigma, multiculturalism, multicultural competence, cultural humility, discrimination, conservatism)

| - Yes, include   (select article for full-text screening) | - Unsure   (select article for full-text screening) | - No, exclude ( screen next article) |
| --- | --- | --- |

## Supplementary Figure 2. Full Text Screening Tool.

1. Do the Method or Result section describe the measurement and resulting analysis of facets of openness to experience?

| - Yes ( proceed) | - Unsure ( proceed) | - No, exclude ( next article) |
| --- | --- | --- |

1. Do the Method or Result section describe the measurement and resulting analysis of tolerance^1^ or prejudice^2^?

| - Yes, ( proceed) | - Unsure ( proceed) | - No, exclude ( next article) |
| --- | --- | --- |

1. Does the study investigate one of the following?

Prejudice or Tolerance

| - Key terms and definition | - Similar terms: | Unrelated terms: |
| --- | --- | --- |
| Prejudice = a negative attitude (with cognitive, affective, & behavioral components) towards a specific or generalised target group | - Racism, homophobic, stigma (others), xenophobic | - political ideology, political attitude, conservatism, right wing authoritarianism, right wing political orientation, dogmatism, fundamentalism, extremist ideology |
| Tolerance = acceptance of diversity, respect for diversity, and appreciation for diversity. | - xenophilia, openness to diversity, multiculturalism, pluralism | - Liberalism, egalitarianism, universalism |
| Cultural competency = refers to a set of cultural behaviors and attitudes integrated into the practice methods of a system, agency or its professionals, that enables them to work effectively in cross cultural situation | - Multicultural competency, Cross cultural competency |  |

or Tolerance (DV)

Openness facets

Prejudice or Tolerance

Openness facets

Mediating variable X

or

Moderating variable X

Prejudice or Tolerance

Variable X

Openness facets

| - Yes, include article | - Unsure, classify article under ‘Maybe’ | - No, exclude ( screen next article) |
| --- | --- | --- |

^1^Tolerance is defined as an acceptance of diversity, respect for diversity, and appreciation for diversity. Related terms are xenophilia, openness to diversity, multiculturalism, pluralism, universalism. Unrelated terms are liberalism, egalitarianism, identification with all humanity, global citizenship, and color-blindness.

^2^Prejudice is defined as a negative attitude towards a specific or generalized target group. Related terms are racism, homophobic, stigma (others), xenophobic, ethnocentrism. Unrelated terms are political ideology, political attitude, conservatism, right wing authoritarianism, social dominance orientation, dogmatism, fundamentalism, and extremist ideology.
